# The effects of a home-based exercise intervention on elderly patients with knee osteoarthritis: a quasi-experimental study

**DOI:** 10.1186/s12891-019-2521-4

**Published:** 2019-04-09

**Authors:** Hongbo Chen, Xiaoyan Zheng, Hongjie Huang, Congying Liu, Qiaoqin Wan, Shaomei Shang

**Affiliations:** 1 School of Nursing, Peking University, 38 Xueyuan Road, Haidian District, Beijing, 100191 China; 2 The Open University of China, 75 Fuxing Road, Haidian District, Beijing, 100039 China; 3 Institute of Sports Medicine, Peking University Third Hospital, 49 Huayuan North Road, Haidian District, Beijing, 100191 China

**Keywords:** Osteoarthritis, Aged, Exercise, Pain

## Abstract

**Background:**

Knee osteoarthritis (KOA) is common in elderly people, causes pain, loss of physical functioning, and disability. This was a two-arm, superiority, quasi-experimental trial. The aim of this study was to evaluate the effectiveness of a home-based exercise intervention (HBEI) to reduce KOA symptoms and improve the physical functioning of elderly patients.

**Methods:**

A total of 171 elderly patients (60 years of age or older) with KOA were recruited from four community centers. Patients from two community centers were randomly assigned to the intervention group (IG) and the other two centers were randomly assigned to the control group (CG). Participants in the IG received a 12-week HBEI (including four 2-h sessions supervised by a physiotherapist and fortnightly telephone support) combined with health education, while patients in the CG only received health education. The participants and physiotherapists were aware of the group assignment and alternative treatment components, but the study’s hypotheses were not disclosed to the participants. Pain intensity, joint stiffness, lower-limb muscle strength, balance, mobility, and quality of life were measured before and after the intervention by the same blinded assessor.

**Results:**

A total of 171 patients (IG: *n* = 84, CG: *n* = 87) were enrolled. Data were obtained from 141 patients with an average age of 68 (range, 60–86 years) who completed the 12-week study (IG: *n* = 71, CG: *n* = 70). No significant group differences were found in any outcome measures at baseline. At week 12, the pretest/posttest changes 3significant between-group differences in decreases in pain intensity (− 1.60 (CI, − 2.75 to − 0.58)) and stiffness (− 0.79 (CI, − 1.37 to − 0.21)), with the IG exhibiting significantly larger improvements on both measures than the CG. The IG also showed significantly greater improvements on all the secondary outcomes than the CG did.

**Conclusions:**

HBEI may be effective for relieving KOA symptoms, increasing the physical functioning, and improving quality of life in community-dwelling KOA elderly patients. A large randomized controlled trial with long-term follow-up is needed to confirm these findings.

**Trial registration:**

Chinese Clinical Trails Registry number ChiCTR1800017026 (retrospectively registered). Registered 9 July 2018.

**Electronic supplementary material:**

The online version of this article (10.1186/s12891-019-2521-4) contains supplementary material, which is available to authorized users.

## Background

Knee osteoarthritis (KOA) is a common joint disease in elderly persons, which causes pain, loss of function and disability, and reduces quality of life [1], and affects approximately one-third of people over 60 years of age. The prevalence rate of KOA is estimated to be 42.8% in women and 21.5% in men in the elderly Chinese population [2]. The prevalence of symptomatic KOA in the United States is estimated to be 7–33%, with the prevalence increasing with age; the knee is the most common site of symptomatic OA [3]. As there is no known cure for OA, it is especially important to use non-pharmacological treatments to control its progression, relieve symptoms, and improve knee function and quality of life [4].

Exercise therapy, which is one of the most important non-pharmacological treatments, is a safe and low-cost method for treating KOA that has been shown to delay disease progression, relieve pain, and improve knee function [5, 6]. Clinical guidelines recommend it as the primary non-pharmacologic treatment of KOA [7]. Common types of exercise for treating KOA include aerobic exercise (such as jogging, cycling, and swimming) and specific knee exercises, which include resistance exercise (mainly to strengthen muscles around the knee), proprioceptive exercise, and range of motion exercise [4, 6, 8, 9]. Exercise interventions are usually performed under the supervision of physiotherapists in hospitals or physical therapy institutes, and require professional rehabilitation equipment [10–12]. However, one study reported that 44.2% of KOA patients withdrew from an exercise intervention conducted at a hospital due to time and economic pressures [13]. From the perspective of hospitals, moreover, providing long-term rehabilitation exercise for KOA patients is a drain on medical resources [4]. Therefore, it is imperative to transfer the location of rehabilitation exercise from inside the hospital to outside the hospital.

Rehabilitation physicians and researchers are increasingly recognizing the value of home-based exercise, which is a time-efficient and convenient treatment modality for patients with chronic diseases such as KOA [14]. It can performed by patients individually at home, unsupervised, and without professional equipment [15]. The goal of home-based and hospital-based exercise is the same; that is, to relieve pain and improve function by strengthening lower limb muscles, improving neuromuscular control, and range of joint motion in the affected knees [15, 16]. The difference is that a home-exercise program needs to be easier to learn and safer to do than a hospital program, especially for elderly patients with KOA, because it is not supervised by a physiotherapist. Several current studies of home-exercise programs for patients with KOA have initially confirmed their feasibility and effectiveness [17–19], but the exercise programs used in each study were quite different and the number of studies is small. Moreover, no one has yet conducted home-exercise interventions in China.

This study aimed to evaluate the effectiveness of a program of home-based exercise intervention (HBEI) for older patients with KOA. Our hypotheses were that an HBEI would relieve patients’ symptoms at week 12, including pain and stiffness, and improve their physical functioning and quality of life, compared to a health-education intervention.

## Methods

### Study design

This was a two-arm, superiority, quasi-experimental trial that was assessor-blinded and conducted in Beijing. The end-point for the analysis of the outcomes was 12 weeks after the baseline assessment. The study’s Chinese Clinical Trails Registry number is ChiCTR1800017026.

### Setting and participants

We recruited community-dwelling elderly patients with KOA from four community centers in Beijing via print and social-media advertisements. The study was conducted at the community- health centers of these four communities. After community nurses recruited elderly individuals from the community centers who had been diagnosed with KOA, the doctors screened them, according to the inclusion and exclusion criteria, and determined who could participate in the study. The inclusion criteria were: being 60 years of age or older; experiencing knee pain on most days of the past month; average knee pain in the last week between 3 and 7 on an 11-point numeric rating scale (NRS); and having intact cognitive functioning, as indicated by a Short Portable Mental Status Questionnaire score of 8–10 (The range of score for this questionnaire is 0–10) [20]. The exclusion criteria were: having had joint replacement surgery or arthroscopic surgery on the affected side of the knee; other surgery on lower limbs within the past 6 months; severe deformity of lower limbs (e.g., knee varus or knee valgus); having health problems that can easily induce adverse events during home exercise, such as uncontrolled high blood pressure, a myocardial infarction, cerebral infarction, unstable angina, arrhythmia, severe vision problems, or neurological dysfunction. Then, the participants gave their informed consent and completed the baseline assessments.

### Randomization and blinding

We performed randomization on a community level rather than an individual level to avoid contamination effects among participants within a community. An independent researcher, who was not involved in the treatment or assessments, used the random number function of Excel to generate randomization sequences. The random numbers were placed in opaque envelopes, which were subsequently opened by study staff, who knew the community allocation, in sequence after community recruitment. Patients from two community centers were randomly assigned to the intervention group and patients from the other two centers were randomly assigned to the control group. Participants and physiotherapists were unblinded to group assignment and were aware of the alternative treatment components, but the study’s hypotheses were not disclosed to the participants. The assessor and statistician were blinded to participant allocation.

### Intervention condition

Participants in the intervention group attended four 2-h sessions conducted by physiotherapists over 12 weeks (weeks 0, 2, 4, and 6). Each session, which was conducted in groups of 6–12 patients, included an hour for health education and an hour for exercise. Participants also performed home-based practice during the study. They were instructed to complete a home-exercise diary in order to remind them to do their home exercises. Research assistants telephoned participants at weeks 1, 3, 5, 7, and 9 to discuss their progress and adherence to the home program, and encouraged them to adhere to the exercise program.

Health education involved four modules delivered by physiotherapists that covered the concepts of clinical manifestations, risk factors, clinical examination and diagnosis, treatment and nursing care for KOA, the benefits of exercise, the home environment, and information about daily care for KOA. Each participant in the intervention group received a paper version of material titled “Health Knowledge and Home Exercise Guide for KOA” in order to consolidate their memory of health knowledge and exercise programs (for details, see the Additional file 1).

The exercise program, which was created based on a literature review, clinical practice, and consultation with experts during our previous studies, aimed to increase lower-limb muscle strength and balance, relieve pain, and reduce knee stiffness. Each exercise session involved isometric contractions of the quadriceps, supine straight-leg lifts, leg lifts in the prone position, resistance knee extension, resistance knee flexion, passive knee flexion, passive knee extension, and shifting the center of gravity (left and right/before and after). Nine home-based exercises for individuals with KOA (Table 1) were recommended with an exercise prescription of 30–40 min per day at least 3 days per week. Not all participants performed the same exercise program; the physiotherapists developed personalized plans for the patients based on their physical functioning and knee symptoms.

**Table 1:** Details of home-based exercises

Exercises	Details
Isometric contractions of the quadriceps	1. Sitting or lying down, legs relaxing; 2. Tight the thigh muscles on one side with maximum strength, keep it for 5 s, and relax for 2 s. Repeat 10 times for 1 group and practice 10 groups in succession; 3. Relax this leg and repeat the above action on the other side; 4. Exercise alternately 3 to 5 times with both legs.
Supine straight-leg lifts	1. Lie on the back, stretch knees; 2. One leg is flexed to support the bed surface, the other leg is raised to the heel, about 20 cm away from the bed, held for 5seconds, put down for 5 s, repeat 10 times; 3. Relax this leg and repeat the above action on the other side; 4. Exercise alternately 3 to 5 times with both legs.
Leg lifts in the prone position	1. Lie face down, stretch knees; 2. Lift one leg back to the toe, about 20 cm away from the bed, held for 5 s, put down for 5 s, repeat 10 times; 3. Relax this leg and repeat the above action on the other side; 4. Exercise alternately 3 to 5 times with both legs.
Passive knee flexion	1. Sit on the bed; 2. Hold your hands on one side of the ankle, slowly and forcefully hold the leg to the chest to maximize knee flexion, keep60 s; 3. Relax this leg and repeat the above action on the other side; 4. Exercise alternately 2 to 3 times with both legs.
Passive knee extension	1. Sit on the bed; 2. Put one side of the foot pad 8~10 cm high; 3. Apply light weight to the raised knee joint or apply proper pressure by hand for 60 s; 4. Relax this leg and repeat the above action on the other side; 5. Exercise alternately 2 to 3 times with both legs.
Resistance knee extension	1. Sit on the chair or at the bed, tie a 1 kg sandbag to the ankle, keep the upper body straight; 2. Do not move the thighs, lift your calves until the knees are fully extended, hold for 5 s, rest your legs for 5seconds, repeat 10 times; 3. Relax this leg and repeat the above action on the other side; 4. Exercise alternately 2 to 3 times with both legs.
Resistance knee flexion	1. Standing up, tie a 1 kg weight sandbag to the ankle joint, and support the upper edge of the chair; 2. Stand on one leg and pull the calf back to the other leg, flexing the knee as much as possible while keeping the thigh perpendicular to the ground. Hold for 5 s, put your legs down for 5 s, repeat 10 times 3. Relax this leg and repeat the above action on the other side; 4. Exercise alternately 2 to 3 times with both legs.
Shifting the center of gravity (left and right)	1. Stand up and support a table with a height of 70~80 cm and open the feet; 2. Keep your knees upright, slowly move the center of gravity to the left, and gradually lower your right heel; 3. Keep your knees upright, slowly move the center of gravity to the right, and gradually lower your left heel; 4. Repeat the above action for 3 min.
Shifting the center of gravity (forwards and backwards)	1. Stand up and support a table with a height of 70~80 cm and take one step forward on one side; 2. Keep the knees upright, slowly move the center of gravity forward, and the heel of the hind foot gradually leaves the ground; 3. Keep the knees upright, slowly move the center of gravity backwards, and the forefoot gradually leaves the ground; 4. Repeat the above action for 3 min.

### Control condition

Participants in the control group received the same number of health-education sessions, telephone follow-ups, and the same paper materials as the participants in the intervention group, but their health education and paper materials did not include exercise-related information. Their health face-to-face education sessions were also conducted in groups of 6–12 patients. During the telephone follow-up, the research assistants asked about the patient’s condition, answered the patient’s questions, and told the patient to protect the knee in accordance with the health-education content about daily care. The control group did not receive any guidance about home-based exercise.

### Training of physiotherapists, research assistants, and the assessor

The physiotherapists, who worked in a general hospital and had ≥5 years of musculoskeletal clinical experience, underwent at least 4 h of training about programs of HBEI. The research assistants and the assessor were medical undergraduates who received at least 8 h of training regarding their respective tasks. All the training sessions in our study were conducted by the researchers.

### Outcome assessment

The participants were assessed by the same blinded assessor at baseline (pretest) and week 12 (posttest). Personal information was collected using a demographic questionnaire developed for this study, which included questions about age, gender, height and weight (body mass index [BMI]), ethnic group, marital status, educational level, occupation before retirement, average monthly household income, residence, living arrangement, disease duration, comorbidities, and history of falls within the past year. All the questionnaire used in our study were administered on paper.

### Primary outcome measures

The primary outcomes of the study were pain intensity and joint stiffness related to KOA. Pain intensity and joint stiffness related to KOA were measured by the Western Ontario and McMaster Universities Osteoarthritis Index (WOMAC) [21]. It includes 7 items on pain and joint stiffness that are rated on a 0–4 Likert scale. Higher scores reflect greater pain and stiffness. The internal reliability of the Chinese version of the WOMAC, as measured by Cronbach’s alpha, is 0.67–0.82 for its three subscales and its test-retest reliability, based on the intraclass correlation coefficient (ICC), is 0.82–0.88 for its three subscales [22].

### Secondary outcome measures

The secondary outcomes included the muscle strength of the lower limbs, balance, walking ability, and quality of life. The muscle strength of the lower limbs was measured by the Five-Times-Sit-to-Stand Test (FTSST), in which participants rise from a chair and return to a seated position as quickly as possible with their arms folded across their chests. The time to complete five repetitions was recorded for two separate trials, with a 1 min rest between each trial. The mean of the two trials was computed and used in the analysis [23].

Balance was measured using the Timed Up and Go test (TUG). The TUG assesses the length of time it takes participants to get up from a standard height chair, walk 3 m, turn and return to the chair, and sit down again [24]. Walking ability was measured using the Six-Minute Walk Test (6MWT). This test measures the distance an individual is able to walk in 6 min on a 30 m, hard, flat, indoor surface. Standardized verbal encouragement is allowed to be provided at 1 min intervals and during rest [25].

Quality of life was measured by the Arthritis Impact Measurement Scales 2 - Short Form (AIMS2-SF), which is a self-assessment scale specifically for arthritis patients [26]. It consists of 26 items measured on a 5-point Likert scale. As we were assessing the quality of life of elderly patients with KOA in our study, we deleted two items about the hand and arm and two items about work, resulting in a 19-item scale that included seven items from the body dimension, three items from the symptom dimension, five items from the emotional dimension, and four items from the society dimension. The range of possible scores was 19–95. Internal consistency, as measured by Cronbach’s alpha, was 0.65–0.83 [27].

We designed a questionnaire to assess the patient’s exercise compliance. The questionnaire contained four items, including “exercise at least 3 times per week,” “reach the desired amount of exercise each time,” “guarantee the quality of each action,” and “total home-exercise time is 30–40 minutes each time.” The items were rated on a 4-point scale, in which 1 = “cannot do it at all” and 4 = “can do it completely.” Exercise compliance rate = actual score/theoretical maximum score × 100%. The patients were divided into three categories, based on their exercise compliance rate: high (≥75%), medium (75–50%), and low (≤50%).

### Sample size

The primary outcome was the change in the pain and joint stiffness dimension of the WOMAC between the experimental group and control group at the end of the treatment (12 weeks). Assuming a mean difference (and SD) between the groups based on the results of relevant research on exercise interventions [28], power analysis was conducted with α = 0.05, β = 0.90, and intervention and control groups of equal sample size. Power Analysis and Sample Size (PASS 2008) software estimated that 69 patients were needed per group. Given a projected dropout rate of 15%, we aimed to include 80 patients per group.

### Statistical analysis

The data were analyzed using SPSS version 23.0 (IBM Corporation, Armonk, NY, USA). Descriptive statistics, including means and standard deviations (SDs), or medians and the interquartile range (IQR), as well as frequencies and percentages, were used to summarize the demographic and disease characteristics and scores on the FTSST, TUG, 6MWT, and WOMAC. Inferential statistics were used to analyze the data, including the independent *t*-test, Mann-Whitney U test and the general linear model (GLM). As the randomization unit was the communities rather than the study participants, the GLM treated group as a fixed factor and community as a random factor, with baseline scores as covariates.

## Results

**Fig. 1:**
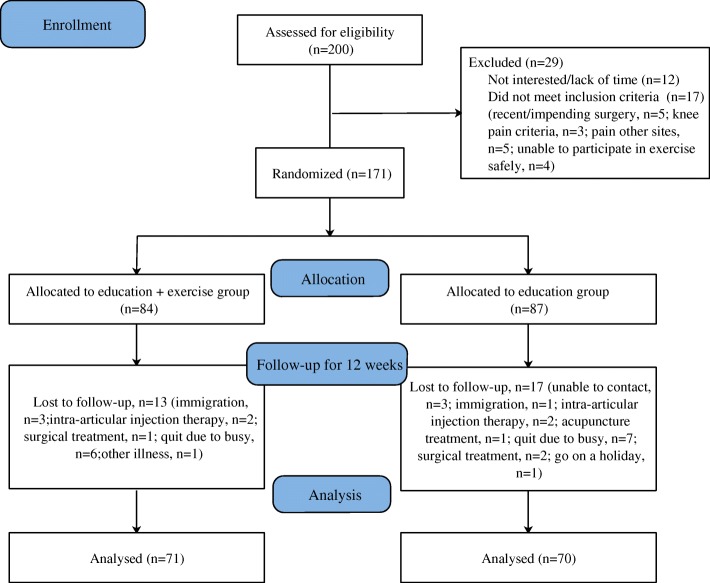
Flowchart of the study participants

The descriptive characteristics of the participants are shown in Table 2. Most of the participants were women (84.4%), married (84.4%), had a junior high-school education (34.0%), had two affected knees (55.3%), and did not use a walker (96.5%). The most common comorbid condition was hypertension (48.2%), followed by diabetes (20.6%), coronary heart disease (27.0%), and osteoporosis (14.9%). About one-third of the participants used analgesics (32%) and cartilage-protective drugs (29%) to relieve pain and other symptoms of KOA. Figure 1 shows a flowchart of numbers of patients at different points in the study. Of the 200 patients from four community centers, 29 (14.5%) were ineligible or did not wish to participate, and 171 were enrolled. The treatment groups were similar at baseline with respect to demographics, clinical characteristics, and amount of therapy (Table 2). Thirty of the 171 patients were lost to follow-up (17.5%). The patients who were lost to follow-up had similar baseline characteristics and outcomes as the patients who remained in the study (data not shown). The overall rate of attending the exercise-class sessions was 82.5%. At 12 weeks, there were 51 participants (71.8%) with good compliance (compliance rate ≥ 75%), 16 (22.6%) with moderate compliance(compliance rate 50–75%), and four (5.6%) with poor compliance (compliance rate ≤ 50%).

**Table 2:** The demographic characteristics of the recruited sample at baseline

Characteristic	Total (*n* = 141)		Intervention (*n* = 71)		Control (*n* = 70)		*P*-value
	*N*	(%)	*n*	(%)	*n*	(%)	
Age - Mean (SD), y^b^	68.9	(7.35)	68.9	(7.78)	68.8	(6.96)	0.963
Gender^a^							
Male	22	(15.6)	12	(16.9)	10	(14.3)	0.669
Female	119	(84.4)	59	(83.1)	60	(85.7)	
Body mass index - Mean (SD), kg/m^2^^b^	25.2	(3.48)	25.0	(3.45)	25.4	(3.51)	0.565
Symptom duration - Mean (SD), y	6.4	(8.52)	6.7	(9.39)	6.0	(7.60)	0.664
Level of education^a^							0.524
Primary school or less	22	(15.6)	12	(16.9)	10	(14.3)	
Junior high school	48	(34.0)	25	(35.2)	23	(32.9)	
High school	31	(22.0)	12	(16.9)	19	(27.1)	
College graduate and above	40	(28.4)	22	(31.0)	18	(25.7)	
Marital status^a^							0.669
Single	22	(15.6)	12	(16.9)	10	(14.3)	
Married	119	(84.4)	59	(83.1)	60	(85.7)	
Number of affected knees ^a^							0.207
One	63	(44.7)	28	(39.4)	35	(50.0)	
Two	78	(55.3)	43	(60.6)	35	(50.0)	
Uses a walker^a^							0.637
Yes	5	(3.5)	2	(2.8)	3	(4.3)	
No	136	(96.5)	69	(97.2)	67	(95.7)	
Comorbid conditions^a^							
Hypertension							0.553
Yes	68	(48.2)	36	(50.7)	32	(45.7)	
No	73	(51.8)	35	(49.3)	38	(54.3)	
Diabetes							0.560
Yes	29	(20.6)	16	(22.5)	13	(18.6)	
No	112	(79.4)	55	(77.5)	57	(81.4)	
Coronary heart disease							0.743
Yes	38	(27.0)	20	(28.2)	18	(25.7)	
No	103	(73.0)	51	(71.8)	52	(74.3)	
Osteoporosis							0.105
Yes	21	(14.9)	14	(19.7)	7	(10.0)	
No	120	(85.1)	57	(80.3)	63	(90.0)	
Current drug use							
Analgesics							0.654
Yes	32	(22.7)	15	(21.1)	17	(24.3)	
No	109	(77.3)	56	(78.9)	53	(75.7)	
Cartilage protection drugs							0.067
Yes	29	(20.6)	19	(26.8)	10	(14.3)	
No	112	(79.4)	52	(73.2)	60	(85.7)	

^a^Chi-square or Fisher’s Exact tests were used

^b^Independent samples *t*-test was used

### Pain intensity and joint stiffness

No significant group differences were found in pain intensity or joint stiffness at baseline. The baseline (pretest) average pain score on the WOMAC was 7.34 (SD 3.36) in the intervention group and 7.19 (SD 4.48) in the control group (see Table 3). The median baseline stiffness score on the WOMAC was 2 (IQR 0,3) in the intervention group and 2 (IQR 1,4) the control group. At week 12 (the posttest), however, the average pain score of the intervention and control groups had decreased to 4.28 (SD 3.30) and 5.73 (SD 3.54), respectively; the median stiffness score was 1 (IQR 0,3) in the intervention group, but it was still 2 (IQR 1,4) in the control group. The GLM showed that the pretest/posttest changes reflected significant between-group differences in decreases in pain intensity (− 1.60 (CI, − 2.75 to − 0.58)) and stiffness (− 0.79 (CI, − 1.37 to − 0.21)), with the intervention group exhibiting significantly larger improvements in outcomes on both measures than the control group.

**Table 3:** Outcome measures over time by group^b^

	Intervention (*n* = 71)		Control (*n* = 70)		*P*-value‡	F	*P*-value†	Parameter estimate of GLM	95% CI of parameter estimate of GLM
	Baseline (mean ± SD)	12 weeks (mean ± SD)	Baseline (mean ± SD)	12 weeks (mean ± SD)					
Primary outcome									
WOMAC pain	7.34 ± 3.36	4.28 ± 3.30	7.19 ± 4.48	5.73 ± 3.54	0.683	7.575	0.007*	−1.60	−2.75,-0.58
WOMAC stiffness ^a^	2 (0,3)	1 (0,3)	2 (1,4)	2 (1,4)	0.428	7.215	0.008*	−0.79	−1.37, -0.21
Secondary outcomes									
FTSST, s	14.22 ± 3.10	12.13 ± 2.93	14.49 ± 4.10	14.13 ± 4.13	0.669	40.272	<0.001*	−2.82	−3.70, -1.94
TUG, s	13.30 ± 3.14	11.73 ± 1.97	13.02 ± 3.27	12.60 ± 2.81	0.611	21.178	<0.001*	−1.37	− 1.96, -0.78
6MWT, m	408.45 ± 60.54	442.39 ± 49.70	422.86 ± 49.29	417.57 ± 53.04	0.124	12.457	0.001*	29.81	13.11, 46.51
AIMS2-SF total	75.06 ± 10.00	82.00 ± 9.96	76.57 ± 10.62	77.90 ± 9.52	0.433	13.263	<0.001*	5.08	2.32, 7.84
AIMS2-SF-body	27.04 ± 4.50	29.54 ± 3.82	27.94 ± 4.77	28.43 ± 4.29	0.174	8.459	0.004*	1.65	0.53, 2.77
AIMS2-SF-symptoms	10.75 ± 2.41	12.62 ± 1.85	10.89 ± 2.11	11.34 ± 1.78	0.998	11.548	0.001*	1.30	0.54, 2.05
AIMS2-SF- emotional^a^	21 (20, 24)	25 (20, 25)	22 (20, 24)	22 (19.75, 24)	0.486	5.640	0.019*	1.11	0.19, 2.03
AIMS2-SF- society^a^	16 (15, 18)	18 (15. 20)	17 (14, 20)	17 (14.75, 20)	0.316	5.403	0.022*	1.11	0.17, 2.05

‡ Independent *t*-test or Mann-Whitney U Test was used (week 0 intergroup comparison)

† The general linear model was used to analyze differences between the intervention and control groups; community was a random factor; baseline scores were covariates; and the control group was the reference

^a^Median (Interquartile Range)

^b^*FTSST* Five-times-sit-to-stand test, the unit of this outcome measure is seconds, *TUG* Timed up and go test, the unit of this outcome measure is seconds, *6MWT* The six-minute walk test, the unit of this outcome measure is meters, *WOMAC* Western Ontario and McMaster Universities osteoarthritis index, pain (0–20), stiffness (0–8), *AIMS2-SF* Arthritis impact measurement scales 2 - short form, body (7–35), symptom (3–15), emotional (5–25), society (4–20), total (19–95), *CI* Confidence interval, *SD* Standard deviation, *GLM* General linear model. *p* < 0.05 was considered statistically significant

### Muscle strength, balance, and mobility

Table 3 shows the results for muscle strength, balance, and mobility. There were no significant group differences in any of the outcomes at baseline. The average level of muscle strength (IG: 14.22 s vs. 12.13 s; CG: 14.49 s vs. 14.13 s) and balance (IG: 13.30s vs. 11.73 s; CG: 13.02 s vs. 12.60s) increased by week 12 in both the intervention and control groups, whereas mobility (IG: 408.45 m vs. 442.39 m; CG: 422.86 m vs. 417.57 m) increased in the intervention group, but decreased in the control group. The GLM, which controlled for baseline scores and the community factor, revealed the intervention group reported significantly better muscle strength (parameter estimate, − 2.82 s [CI, − 3.70 to − 1.94 s] in the FTSST), balance (parameter estimate, − 1.37 s [CI, − 1.96 to − 0.78 s] in the TUG), and mobility (parameter estimate, 29.81 m [CI, 13.11 to 46.51 m] in the 6MWT) than the control group at week 12.

### Quality of life

Table 3 presents the total score and the scores for each dimension of quality of life, as measured by the AIMS2-SF. There were no group differences at baseline. The quality of life of both the intervention and control groups increased by week 12, as measured by their total scores (IG: 75.06 vs. 82.00; CG: 76.57 vs. 77.90) and body-dimension (IG: 27.04 vs. 29.54; CG: 27.94 vs. 28.43) and symptom-dimension scores (IG: 10.75 vs. 12.62; CG:10.89 vs. 11.34). However, the GLM showed the intervention group had a better quality of life than the control group did at week 12, based on statistically significant between-group differences in their posttest scores, including their total score (parameter estimate, 5.08 [CI, 2.32 to 7.84]), body score (parameter estimate, 1.65 [CI, 0.53 to 2.77]), symptoms score (parameter estimate, 1.30 [CI, 0.54 to 2.050], emotional score (parameter estimate, 1.11 [CI, 0.19 to 2.03], and society score (parameter estimate, 1.11 [CI, 0.17 to 2.05]).

## Discussion

This 12-week quasi-experimental study showed that an HBEI and health education significantly reduced symptoms of KOA (i.e., pain intensity and joint stiffness) in elderly patients, and improved their physical functioning (i.e., lower-limb muscle strength, balance, and mobility) and quality of life, compared to an intervention that only involved health education. Furthermore, the overall rate of attending the exercise-class sessions was 82.5, and 71.8% of the patients in exercise group showed good compliance (compliance rate ≥ 75%) with the exercise program.

The control group also showed improvement on many of the outcomes; we attribute this to the effect of health education and the statistical phenomenon of regression toward the mean. The knowledge about proper knee care provided by the health-education program would be expected to help patients to take appropriate measures to relieve their knee pain, which would eventually improve their symptoms. Regression toward the mean is a statistical phenomenon that occurs when repeated measurements are made on the same subjects or units of observation [29]. Even if the true mean does not change, the result of the repeated measurements may increase or decrease due to regression toward the mean. Nevertheless, we found significant group differences in symptom and functional outcomes by week 12 of the study, in which the intervention group showed significantly greater improvement on all of the outcomes, compared to the control group. Therefore, the HBEI may be effective.

This study used the WOMAC index to evaluate the effectiveness of home-based exercise to improve the symptoms of patients with KOA. The results showed that patients who received the exercise intervention combined with health education reported significantly less pain and joint stiffness than patients who only received health education. Spanish scholars Escalantea et al. [30] conducted a meta-analysis of 33 studies on the effect of exercise on knee pain that was evaluated by the pain subscale of the WOMAC. The results showed that exercise reduced knee pain, which is usually the most common and the earliest symptom of patients with KOA. Initial KOA pain is mild or moderate and intermittent, and it is reduced by resting. Late manifestations of KOA pain are persistent or nocturnal, and severely affect the patient’s daily life [31]. Because of a lack of proper knowledge about health, many elderly people with KOA take inappropriate medications or exercise treatments, which eventually lead to increased pain. Therefore, it is vital to provide effective health education and exercise guidance for elderly patients with KOA. HBEI in our study could have relieved knee pain in patients with KOA for the following reasons [32, 33]: (1) HBEI caused the knee cartilage to be squeezed frequently and the joint fluid to continuously enter and exit the matrix, which promoted cartilage growth and joint repair, and reduced knee pain; (2) Proper home exercise promoted the metabolism of the knee’s blood circulation and inflammatory factors, which in turn, reduced inflammation and decreased pain; and (3) Exercise increased muscle strength in the lower extremities and improved the maximum load and stability of the knee, which reduced wear of the articular cartilage and relieved knee pain.

Patients with KOA suffer from joint stiffness due to pain, damage to the cartilage and joint surface, joint effusion, and synovial adhesions. The results of this study regarding the effect of exercise to reduce stiffness in patient are consistent with those of previous studies [13, 34]. Range of joint-motion training combined with lower-limb muscle strength training promotes the range of motion, cartilage metabolism, and the absorption of joint effusion, and removes inflammatory products, which prevents synovial adhesions and the formation of vasospasm, thereby reducing joint stiffness [32].

Compared to healthy elderly people of similar age, patients with KOA generally have poor physical functioning, which is manifested by insufficient lower-limb muscle strength, poor balance, and decreased mobility. Previous studies have shown that there is a 10 to 60% decrease in the strength of the quadriceps muscle in patients with KOA [35], and that the thickness of the quadriceps tendons decreases significantly as the disease progresses [36]. Decreased knee-muscle strength can lead to insufficient joint stability, which leads to an abnormal distribution of stress in the patellofemoral joint and promotes its deterioration. Rasch et al. [37] believes that the muscle weakness caused by osteoarthritis is related to decreased joint function and increased pain, and that quadriceps training can increase muscle strength and relieve joint pain. Canadian researchers Peeler et al. [38] found that the strength of the quadriceps and hamstring muscles of KOA patients living in the community increased significantly after 12 weeks of training. This is consistent with our findings.

Decreased balance and mobility are the main cause of falls in elderly people [39]. Studies have shown that the risk of falls in KOA patients is 30% higher than that of non-KOA patients of the same age [40]. Falls are serious adverse events for elderly people, which may cause disability or even death. According to Arnold’s research [41], an exercise intervention can improve balance and mobility, and prevent falls by elderly patients with KOA. Exercise can increase the muscle strength of the lower limbs to stabilize the knees and coordinate gait [42]. In addition, an increase in neuromuscular control and enhanced swing-knee height when walking can result in a reduced risk of falls [43].

With the transformation of modern medical models, quality of life has been widely used as a comprehensive indicator of health status in some medical fields, including chronic disease. Evaluating the quality of life of KOA patients makes it is possible to understand not only the physical discomfort caused by the disease, but to understand its psychological and social effects on patients. A 2012 study by French researchers Rat et al. [44], which measured the quality of life of 813 patients with arthritis using the AIMS2-SF, found their quality of life significantly decreased, especially on the three dimensions of body, symptoms, and emotion. A 2017 study by Taiwanese researchers Huang et al. [45] showed that the quality of life of KOA patients was negatively correlated with pain intensity and positively correlated with physical functioning. Given these results, the results of a study by the Turkish researchers Aglamis et al. [28] are particularly valuable because they showed that a 12-week multicomponent program of exercise significantly improved the quality of life of patients with KOA. In our study, the exercise intervention decreased pain intensity and joint stiffness in patients, and increased their physical functioning, including lower-limb muscle strength, balance, and mobility. Therefore, the improvement in patients’ quality of life may due to the improvements in pain intensity, stiffness, and physical functioning. However, since we did not conduct mediation analyses with these variables, we cannot confirm there is a clear relationship between improved quality of life and symptom relief and functional improvement.

Major strengths of our study are that the program of HBEI is highly generalizable, that it is simple to learn, and that it does not require any equipment, which facilitates learning and program adherence. Other strengths that ensured the study’s quality and improved the reliability of the results were: (a) its rigorous training and assessment of the research staff, including the physiotherapists, research assistants, and the assessor; (b) its use of blinded assessments and data analyses; (c) its sample size; (d) the comparability of the groups at baseline; and (e) the high participant-retention rate and program adherence rate.

Our study also has some limitations. Although the study’s hypotheses were not disclosed to the participants, they and the physiotherapists were not blinded to group allocation and were aware of the alternative treatment components. Therefore, the absence of blinding would likely have resulted in an overestimation of the effect of the exercise intervention. Due to limited resources, we did not conduct random assignment of participants to groups; nevertheless, the groups were comparable at baseline. We did not include patients with severe pain (NRS > 7) due to concerns about increasing their symptoms with exercise. Therefore, whether HBEI can reduce pain and improve other health outcomes of patients with severe pain remains to be confirmed. Finally, our intervention lasted only 12 weeks; so, we could not determine the long-term effects of the exercise intervention on the patients’ long-term compliance or outcomes.

## Conclusions

Our results showed that HBEI reduced pain intensity and joint stiffness, increased the muscle strength of the lower limbs, balance, and mobility, and improved the quality of life of elderly patients with KOA living in the community. The program is inexpensive, easy to use, safe, and suitable for being practiced at home. A long-term longitudinal study with randomized groups is needed to verify its long-term effects.

## Additional files

Additional file 1: Health Knowledge and Home Exercise Guide for KOA. (DOC 19710 kb)

Additional file 2: Information on the patients enrolled in the study. This file includes the demographic characteristics and all outcomes of the patients enrolled in the study. (XLSX 43 kb)

Additional file 3: CONSORT 2010 checklist of information to include when reporting a randomized trial. (DOC 216 k

Additional file 4: The TIDieR (Template for Intervention Description and Replication) Checklist. (DOCX 29 kb)

## Abbreviations

6MWT: The six-minute walk test
AIMS2-SF: Arthritis Impact Measurement Scales 2 short form
BMI: Body mass index
CG: Control group
FTSST: The five-times-sit-to-stand test
GLM: General linear model
ICC: Intraclass correlation coefficient
IG: Intervention group
KOA: Knee osteoarthritis
NRS: Numeric rating scale
OA: Osteoarthritis
SD: Standard deviation
TUG: The timed up and go test
WOMAC: Western Ontario and McMaster Universities Osteoarthritis Index
