# Supplementary material for: The effects of a home-based exercise intervention on elderly patients with knee osteoarthritis: a quasi-experimental study
Source: BMC Musculoskelet Disord. 2019 Apr 9;20:160. doi: 10.1186/s12891-019-2521-4 (PMC6456993; doi:10.1186/s12891-019-2521-4)
Supplement: Supplementary file 1 — Health Knowledge and Home Exercise Guide for KOA. (DOC 19710 kb) [file 12891_2019_2521_MOESM1_ESM.doc]

**Health Knowledge and Home Exercise Guide**

**Knee Osteoarthritis**


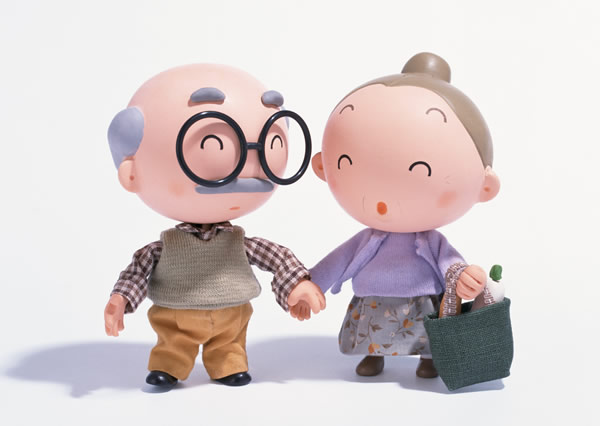


**1.** **What is knee osteoarthritis?**


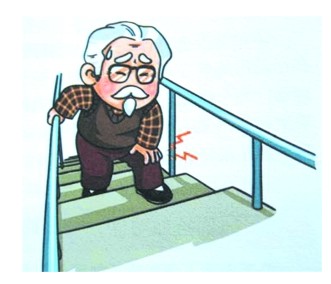
 Knee osteoarthritis (KOA) is the most common joint disease in middle-aged and elderly people, mainly chronic arthritis caused by destruction of articular cartilage and joint degeneration. That is, we often say knee aging, degeneration, bone hyperplasia, bone spurs and so on.

**2.** **What are the symptoms of KOA?**

Initial KOA pain is mild or moderate and intermittent, and it can be reduced by resting. And the pain is most obvious when going up and down the stairs or kneeling and standing up. Joint swelling and tenderness can occur in the knee joint, and there is a feeling of friction or "click" when moving.

In the mid-term KOA, the knee joint pain is aggravated, and the patient may have morning stiffness, that is, the knee joint is stiff and tight after getting up in the morning or after maintaining a posture for a long time, and can be relieved after the activity. Joint stiffness lasts for a short period of time, often from a few minutes to ten minutes, rarely exceeding 30 minutes.

Late KOA showed persistent pain and the patient could not walk because of pain. Severe cases may
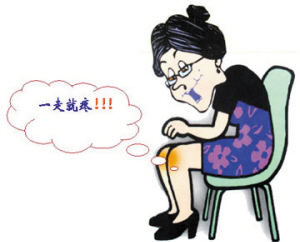
have muscle atrophy and limited mobility. The knee joint is obviously deformed and can be expressed as an "O" leg or an "X" leg.

**3.** **What are the pathogenic factors of KOA?**

1) Age: Incidence increases with age

2) Gender: The incidence of women is greater than that of men

3)Occupation: heavy physical workers, such as porters

4) Obesity

5) Knee joint injury

6) Osteoporosis

7) Others: such as heredity, menopause, cold and humid environment, etc.

**
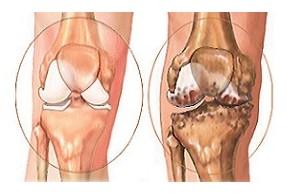
**

正常膝关节

膝关节骨性关节炎

**4.** **How to treat knee osteoarthritis?**

1) **Reasonable diet:** Increase the intake of calcium and vitamin D in the diet

2) **Lose weight:** Keep your weight within the normal range

3) **Cold and warm:** Avoid air conditioning in the summer to face the knee joint; wear warm clothes in winter

4) **Avoid bad posture:** Don't squat for a long time. When you need to sit and stand for a long time, you should change your posture frequently. Do not wear high heels when walking a long distance, wear thick and flexible soft-soled shoes

5) **Reasonable sports:** Including outdoor sports (such as walking, swimming, etc.) and home exercise (such as sports that strengthen the strength of the thigh muscles), in which home exercises are easy to learn, convenient and effective.

6) **Follow the doctor's advice:** such as painkillers, cartilage protective drugs, etc.

7) **Surgical treatment:** Severe cases, such as knee arthroscopy, knee arthroplasty, etc.

**5.** **What are the benefits of exercise for KOA?**

1) **Improve joint load:** Enhance knee strength and maintain knee stability

2) **Increase joint mobility:** Stretch the corresponding tissue to prevent synovial adhesion

3) **Enhance lower limb balance:** Improve physical agility, improve balance, and coordinate gait

4) **Promote cartilage repair and growth:** Promote synovial fluid overflow, provide nutrients for chondrocytes, and promote collagen and proteoglycan synthesis

5) **Anti-inflammatory and pain relief:** Promote blood circulation, help to reduce inflammation, relieve pain and discomfort

6) **Stimulate bone growth:** Increase bone density and prevent osteoporosis

7) **Delay the development of the disease:** Maintain a good state of the body

**Exercise is good for your knees**

**Please exercise reasonably!**

**6.** **How to properly conduct home-exercise?**

1) **Type of exercise:** joint range of motion exercise (R), Lower extremity strength exercise (M), balance function exercise (B) (see Appendix for details)

2) **Movement principle:** Suitable amount, step by step, from simple to complex

3) **Exercise time:** Any leisure time can be selected, you can choose to wake up in the morning or watch TV at night.

4) **Movement frequency:** at least three times a week, 30~40 minutes each time

5) **Exercise intensity:** ①pain or discomfort within the range of tolerance; ②heart rate control during exercise within 50%~75% of maximum heart rate, maximum heart rate = 220-age

Appendix

R-1：Passive knee flexion

| 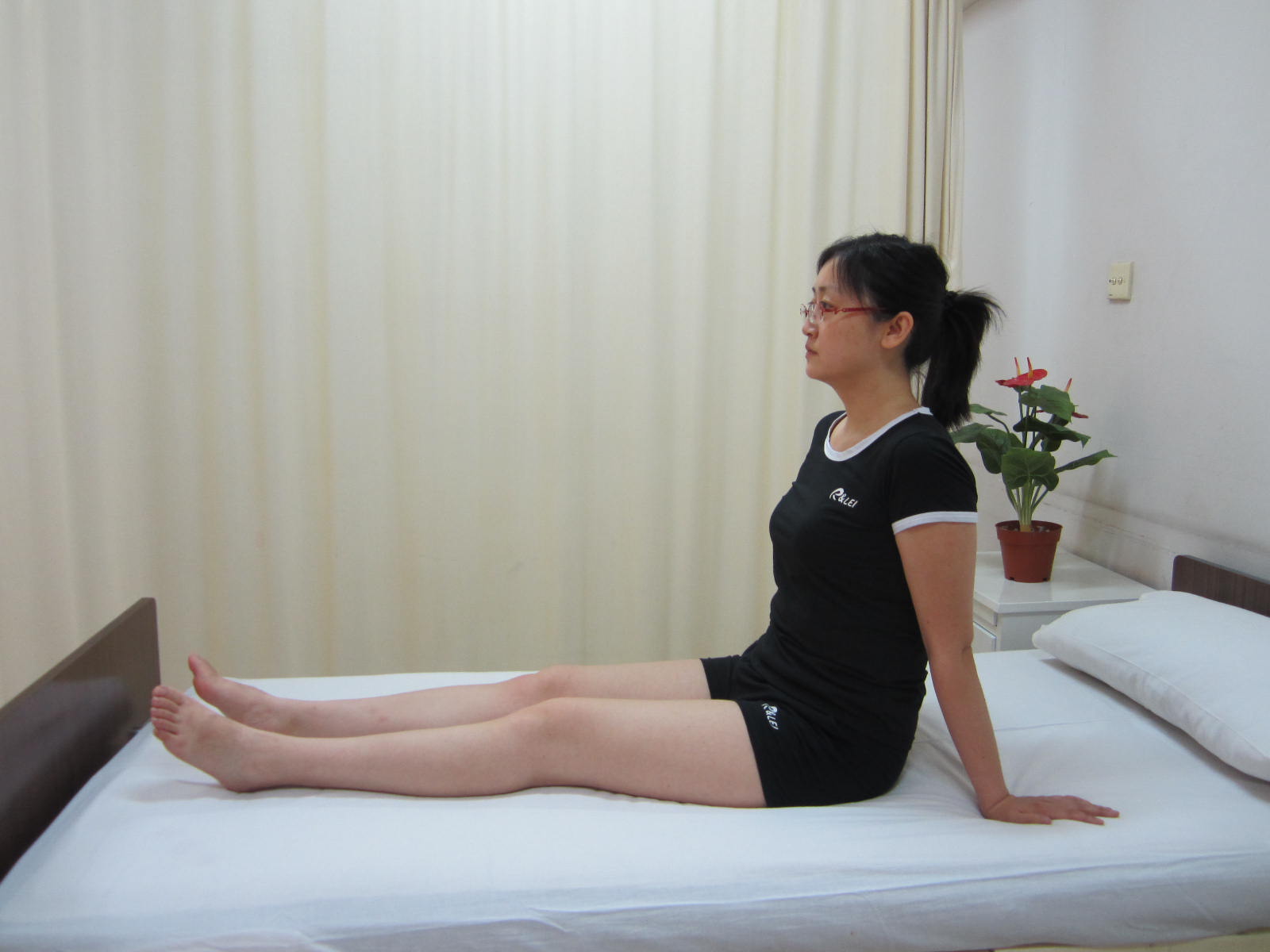  ①Sit on the bed | 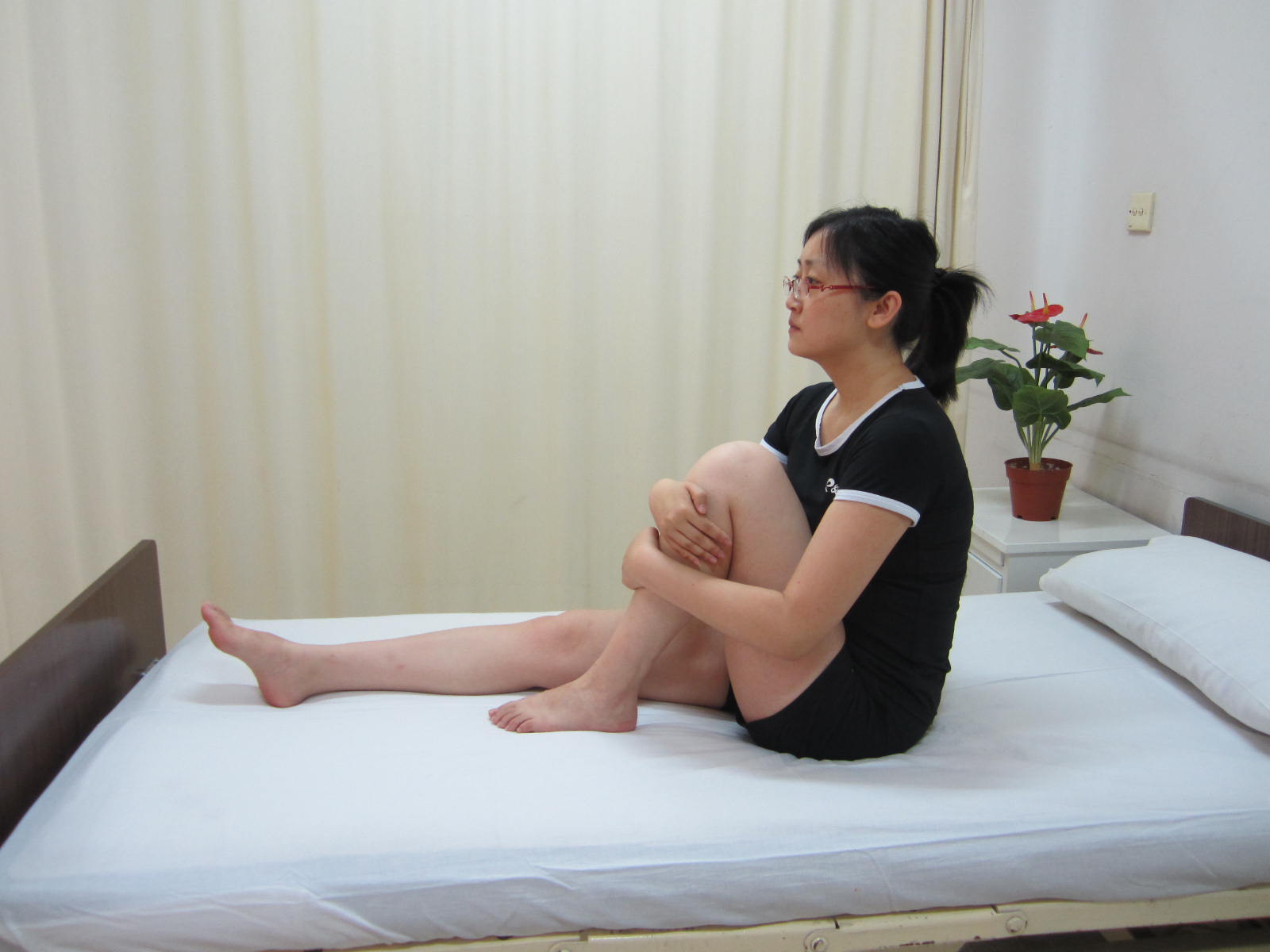  ②Hold your hands on one side of the ankle, slowly and forcefully hold the leg to the chest to maximize knee flexion, keep 60 seconds |
| --- | --- |
| 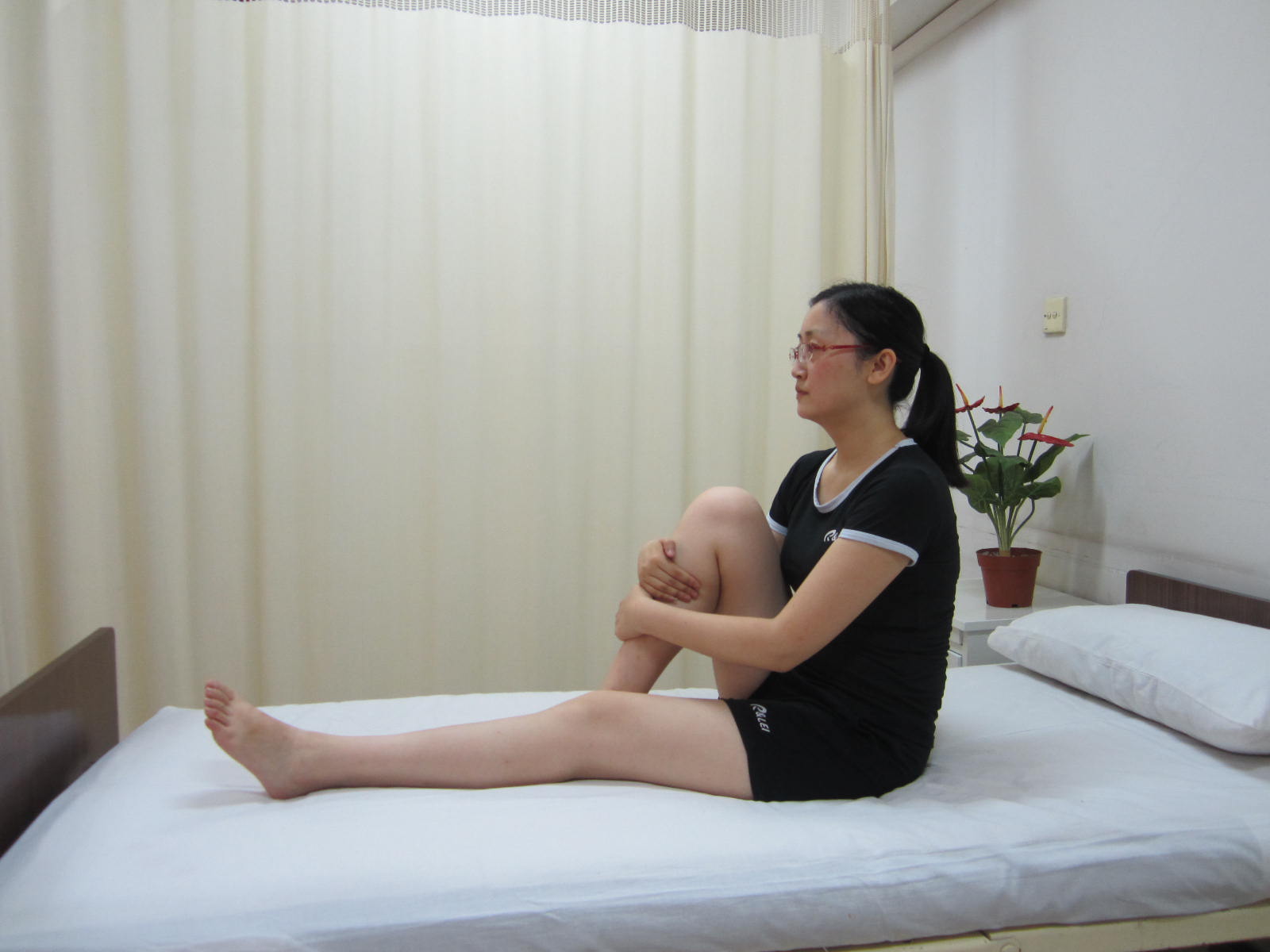③Relax this leg and repeat the above action on the other side | 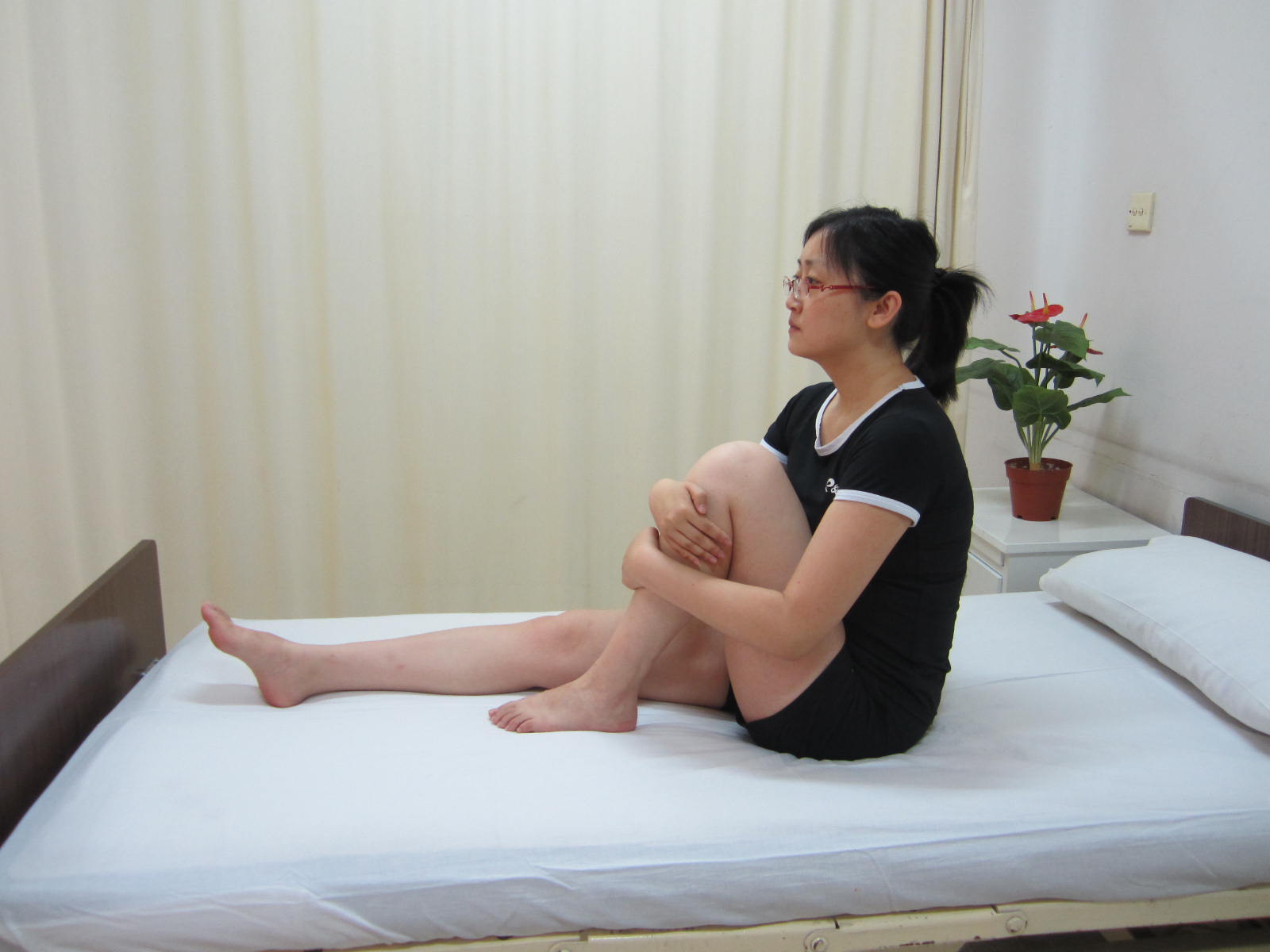  ④Exercise alternately 2 to 3 times with both legs |

R-2：Passive knee extension

| 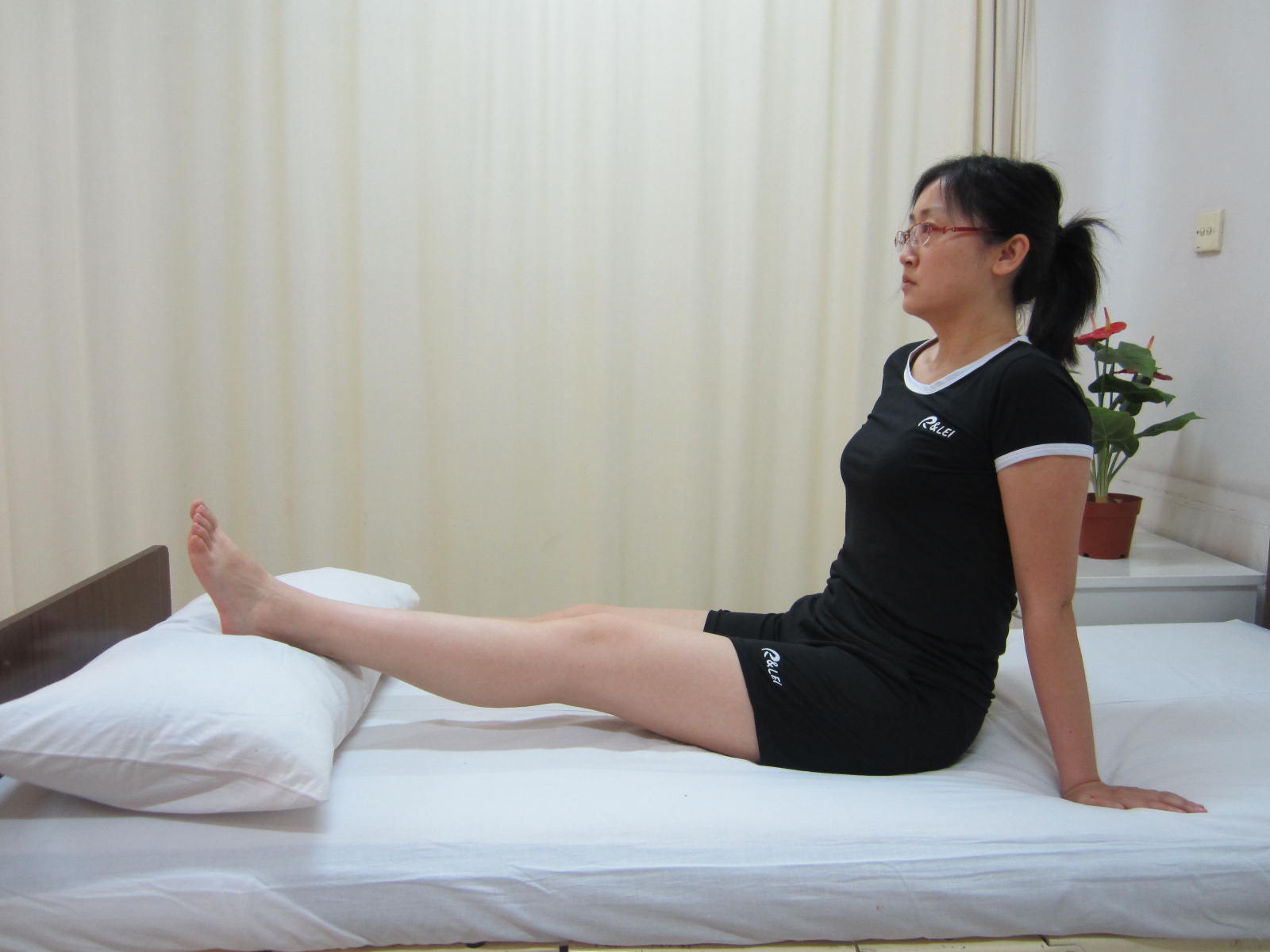①Sit on the bed; Put one side of the foot pad 8~10 cm high | 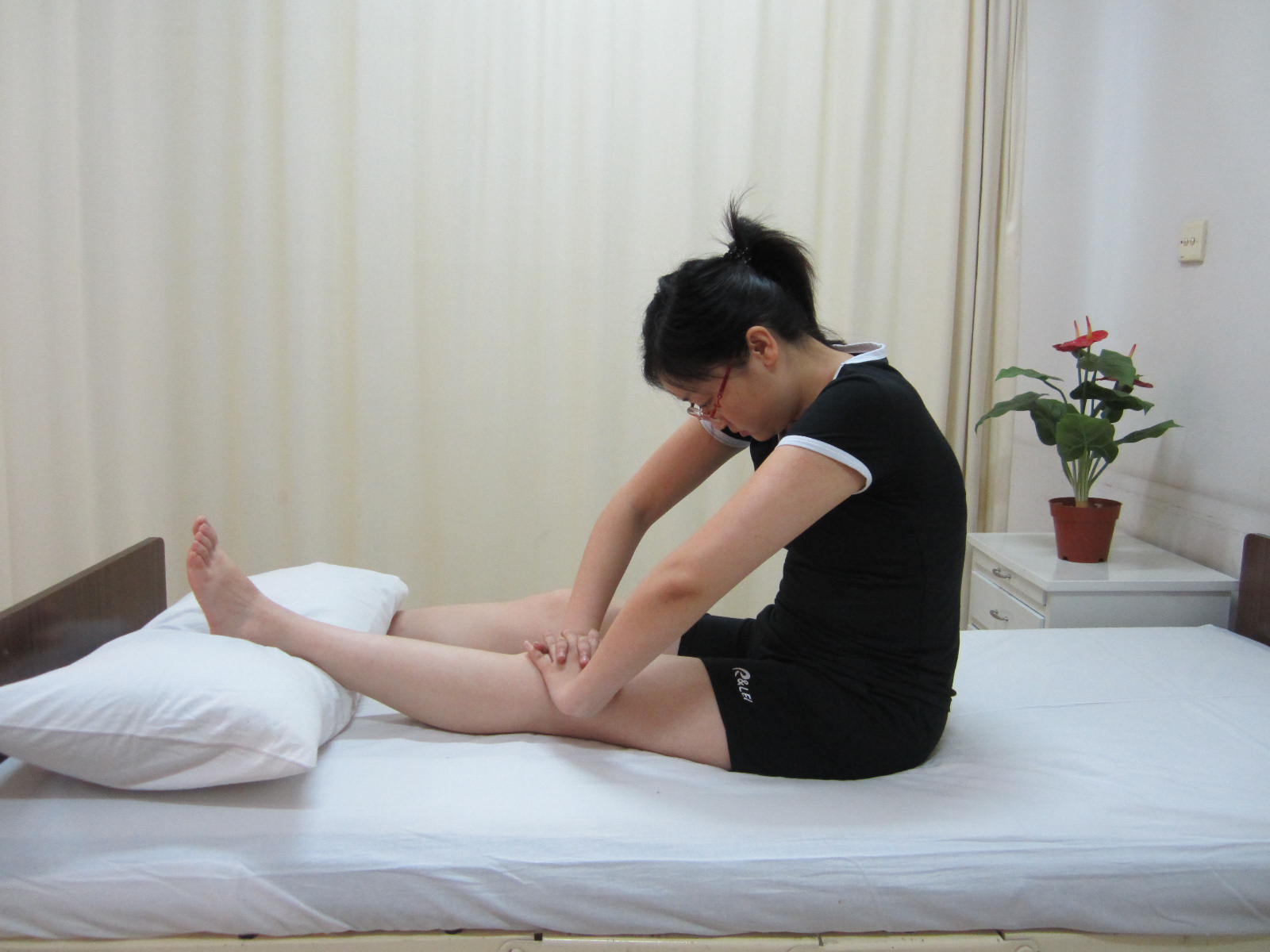  ②Apply light weight to the raised knee joint or apply proper pressure by hand for 60 seconds |
| --- | --- |
| 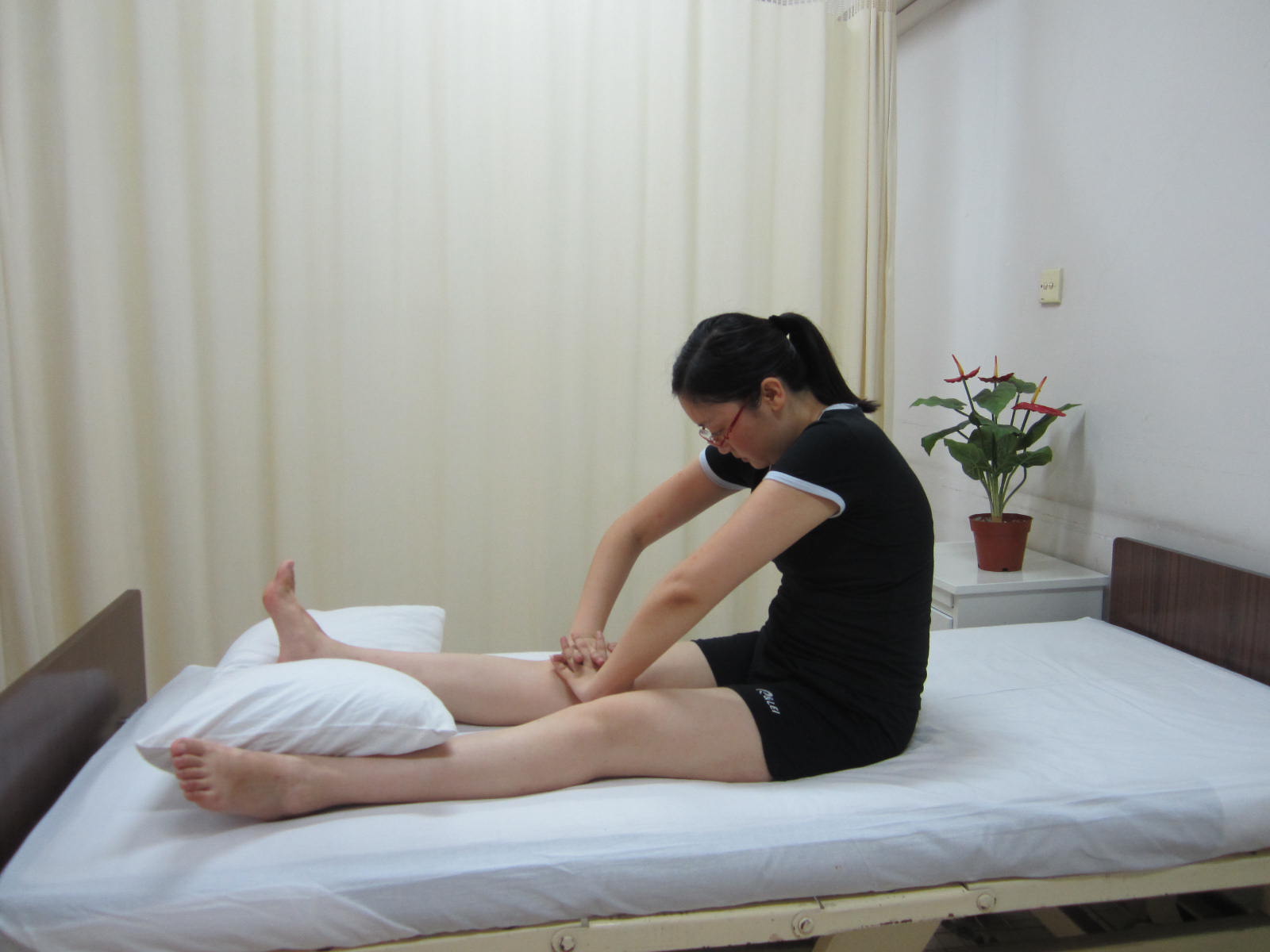③Relax this leg and repeat the above action on the other side | 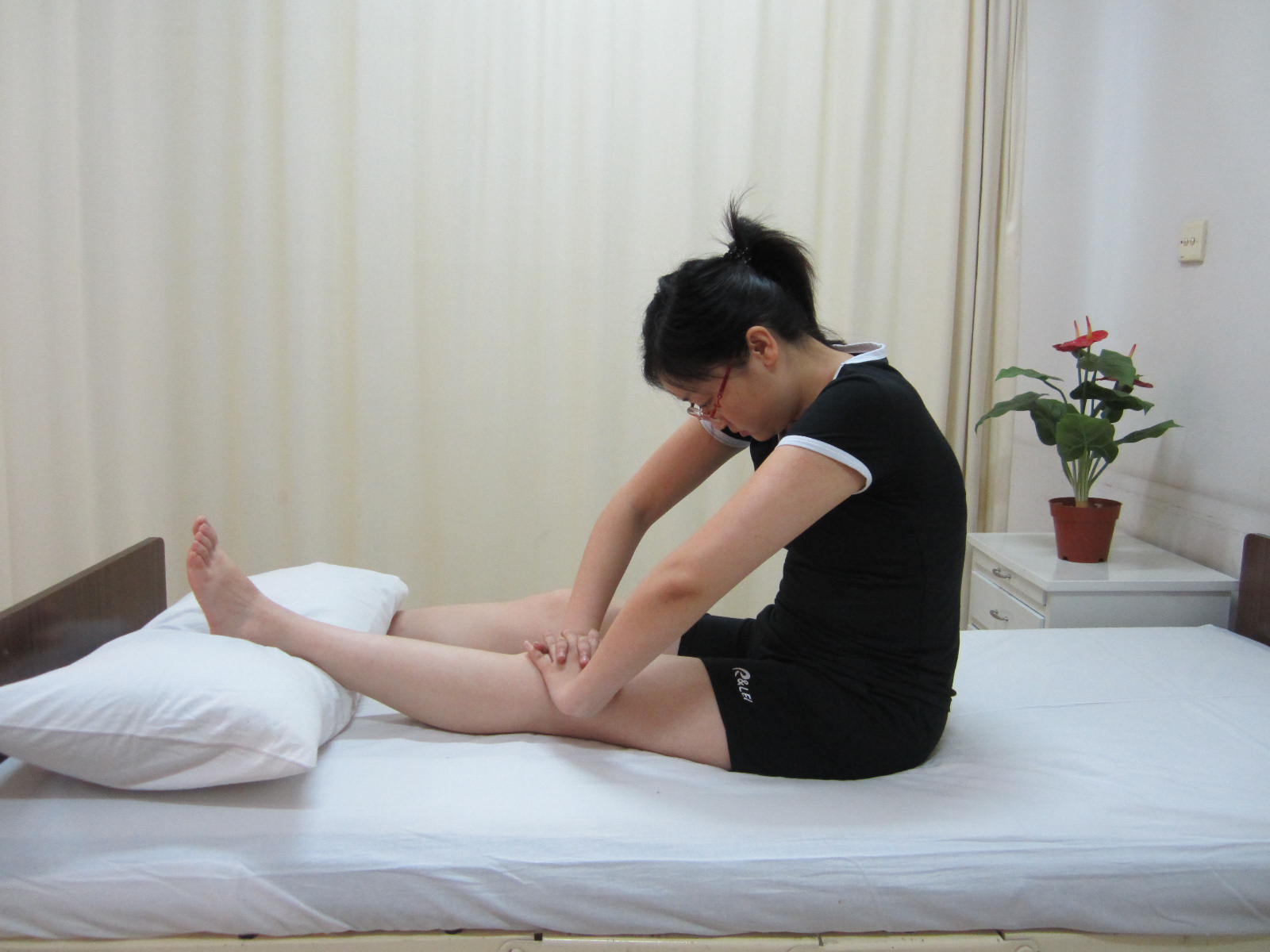  ④Exercise alternately 2 to 3 times with both legs |

M-1：Isometric contractions of the quadriceps

| 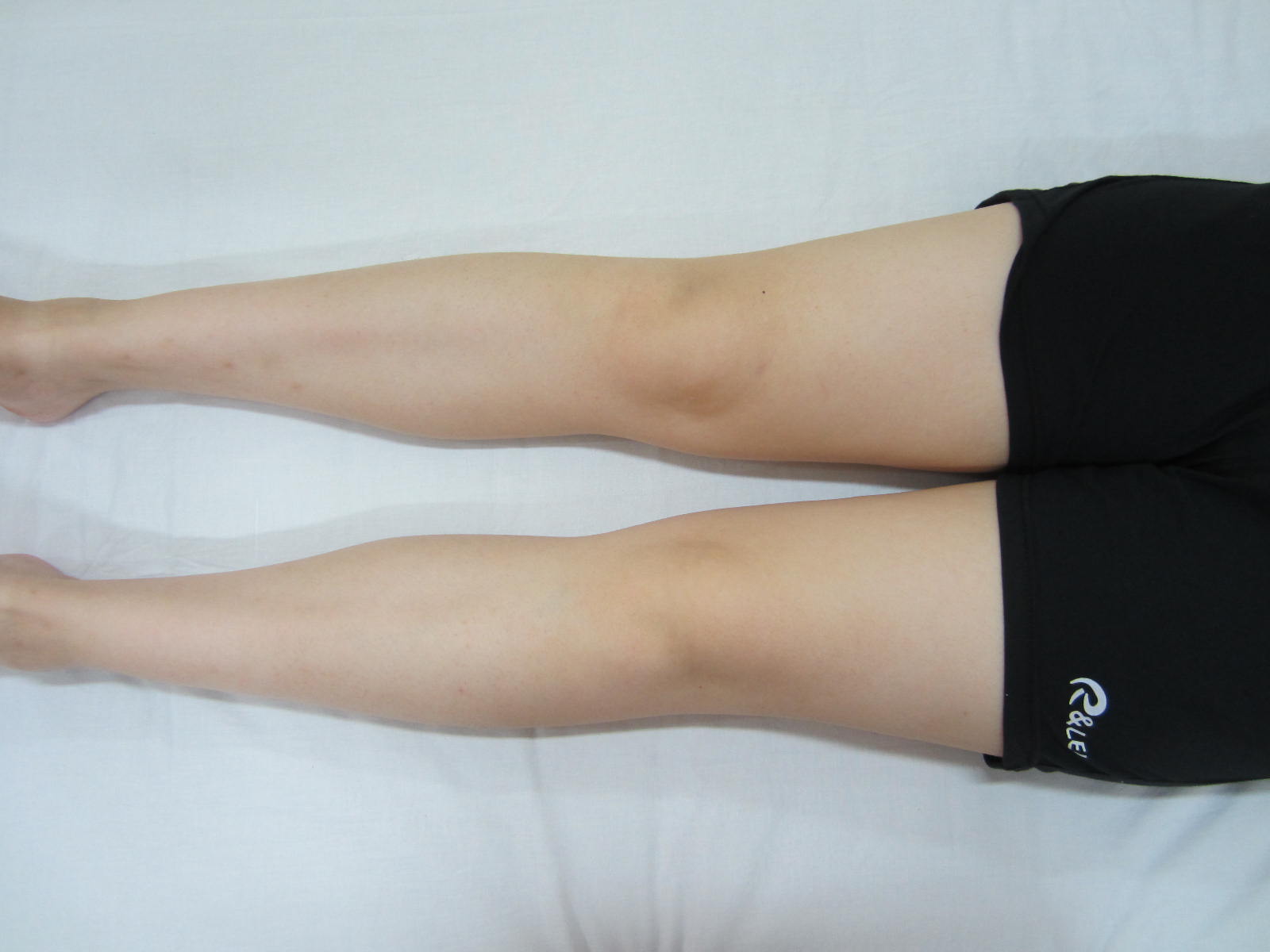  ①Sitting or lying down, legs relaxing | 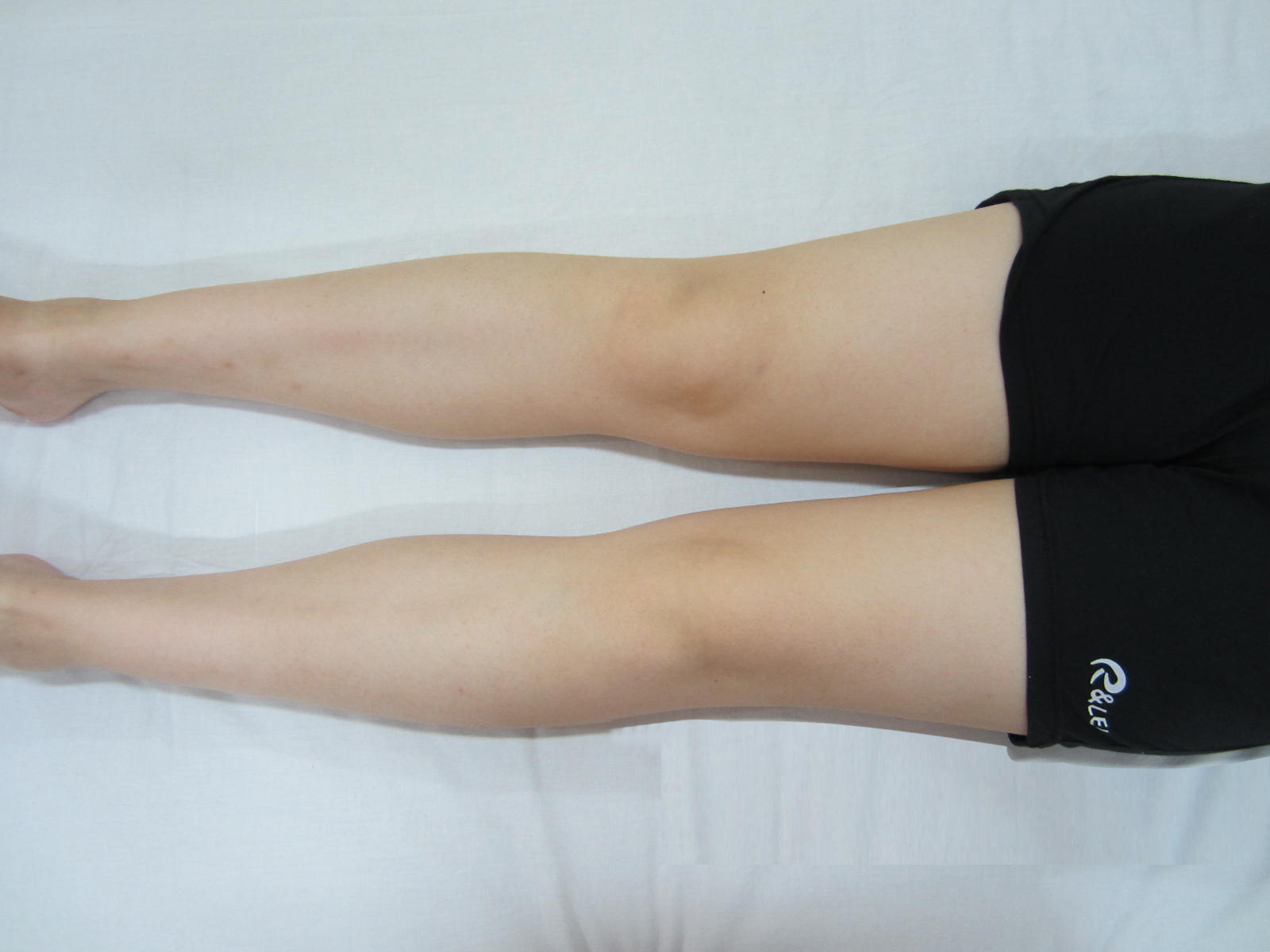②Tight the thigh muscles on one side with maximum strength, keep it for 5 seconds, and relax for 2 seconds. Repeat 10 times for 1 group and practice 10 groups in succession |
| --- | --- |
| 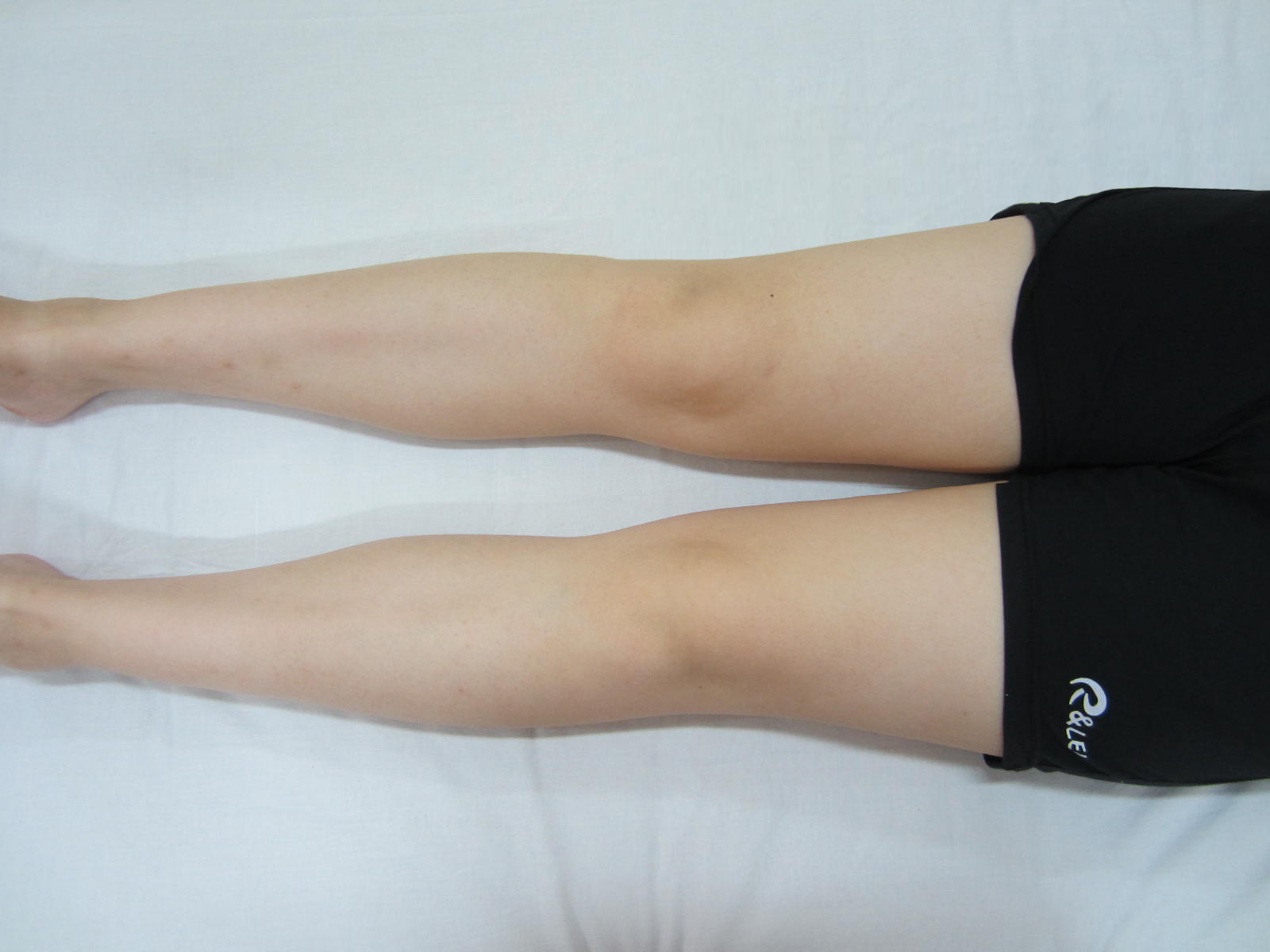  ③Relax this leg and repeat the above action on the other side | 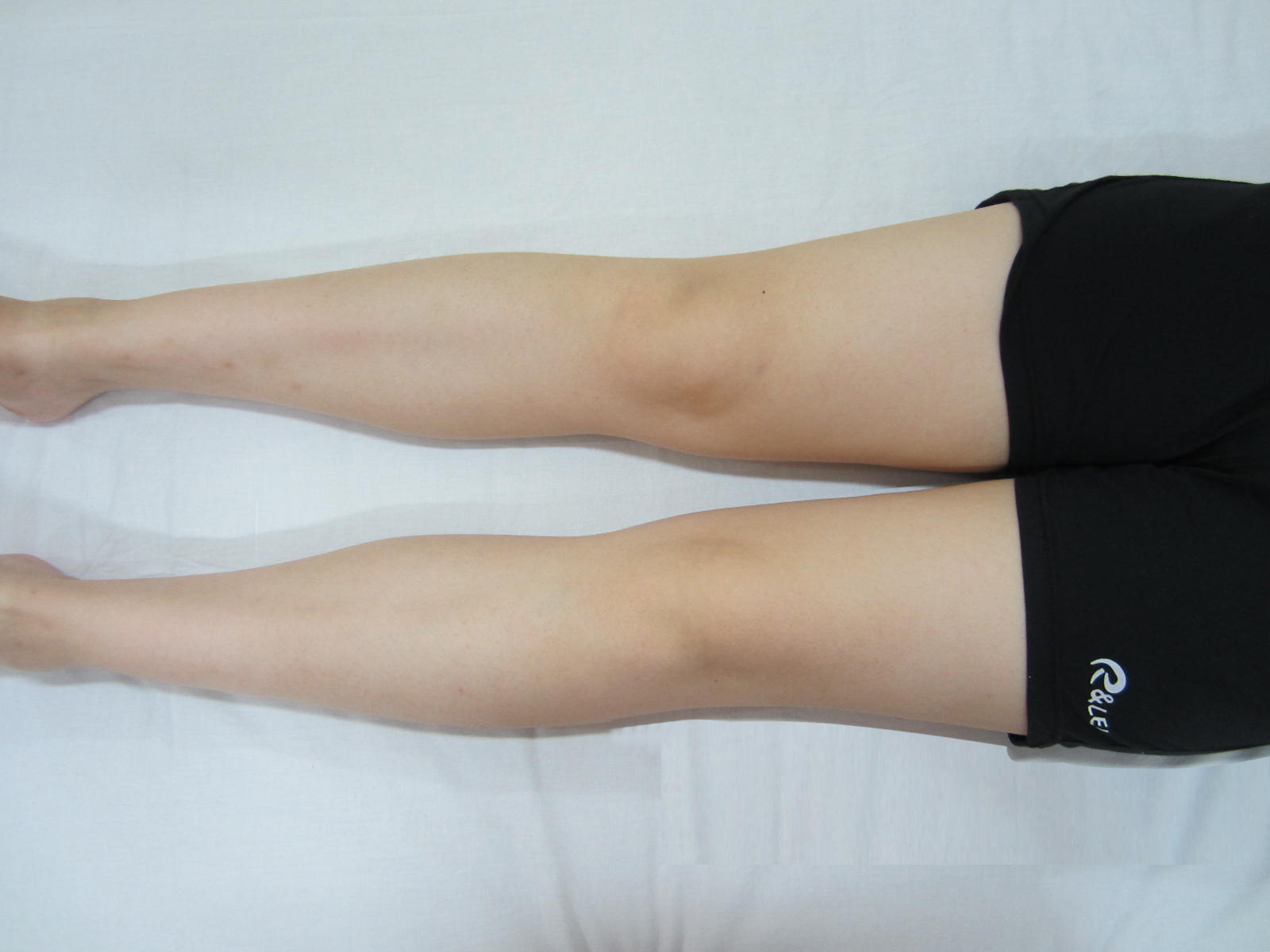  ④Exercise alternately 3 to 5 times with both legs |

M-2：Supine straight-leg lifts

| 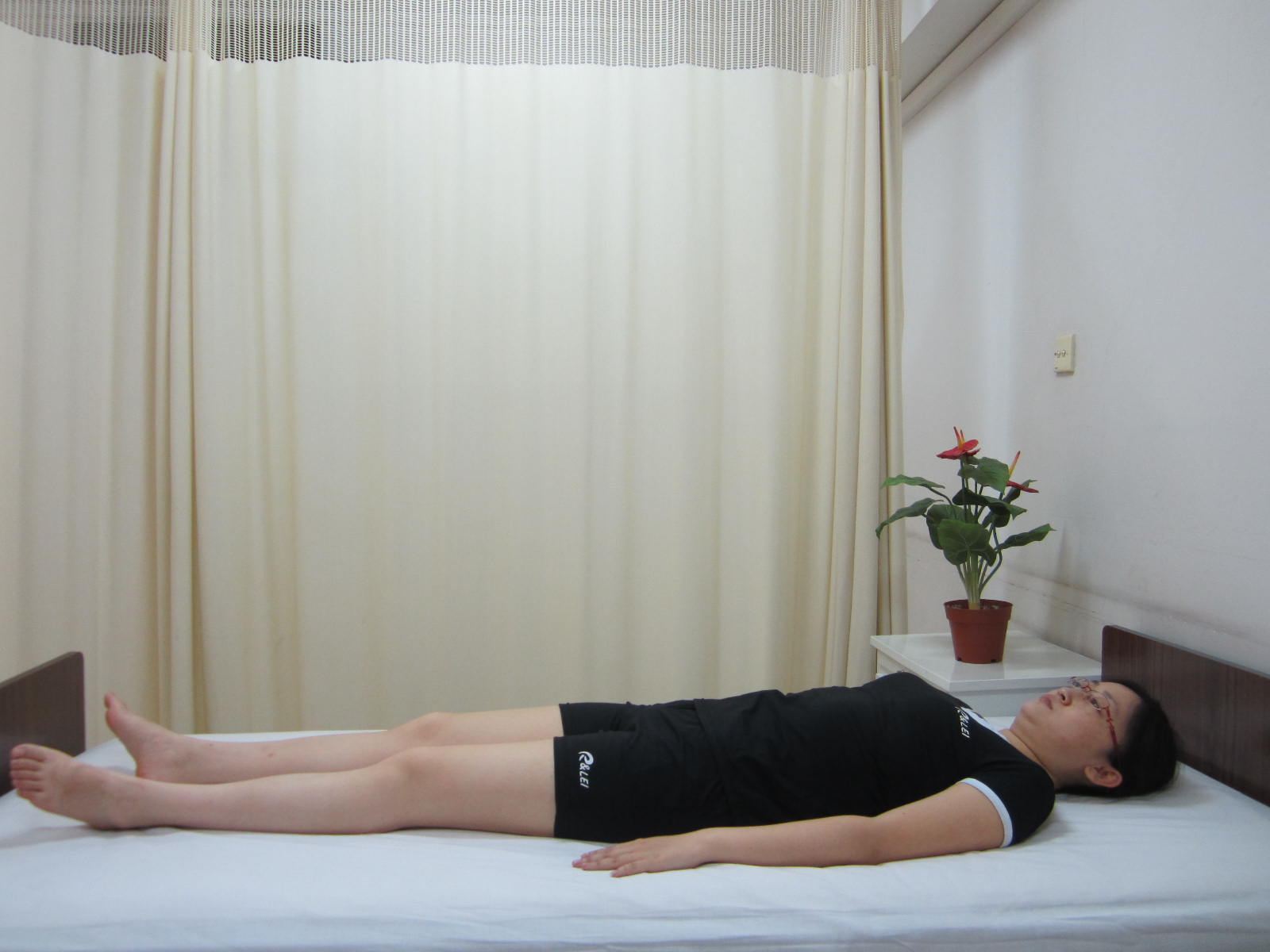  ①lie on the back, stretch knees | 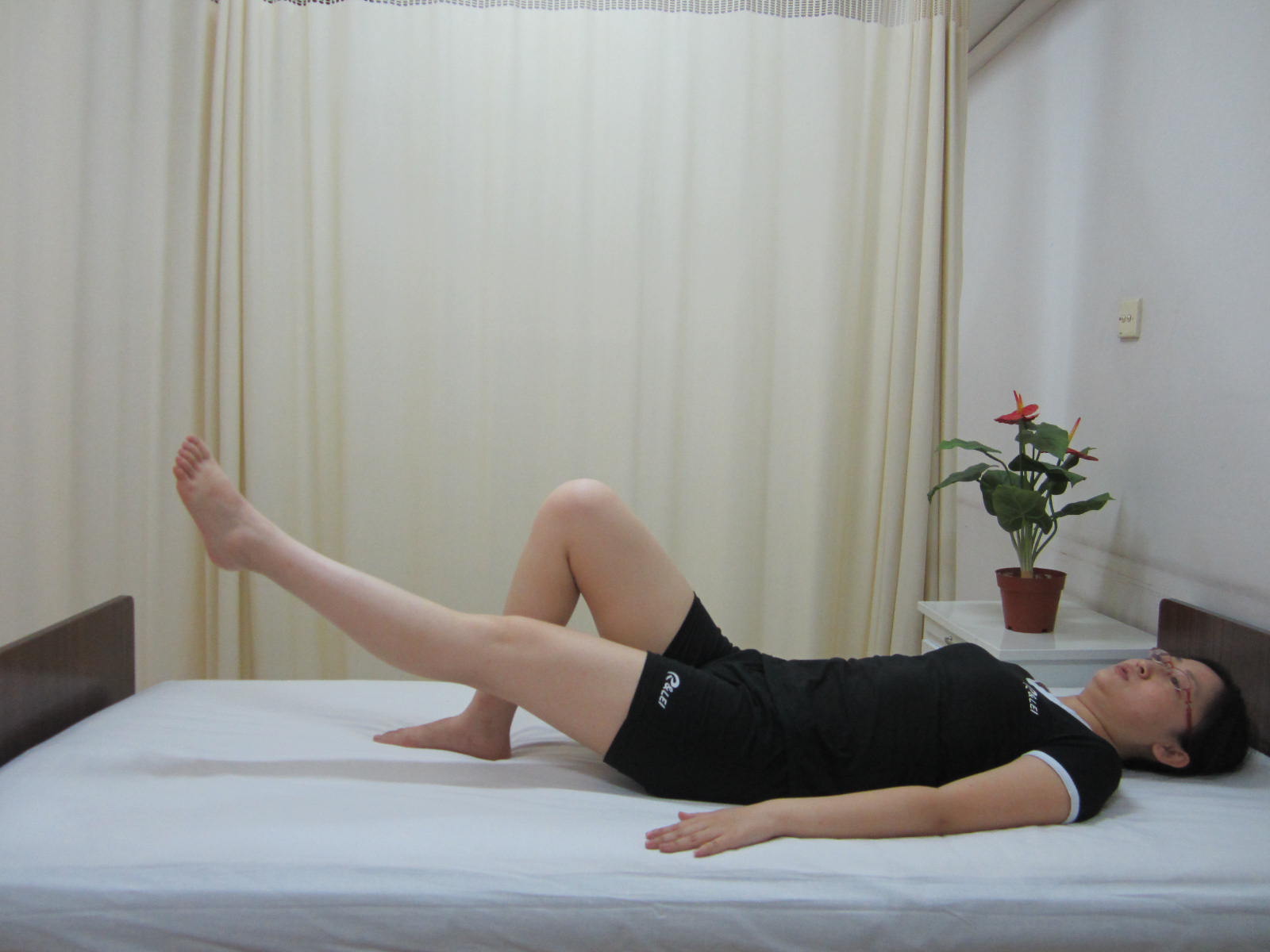  ②One leg is flexed to support the bed surface, the other leg is raised to the heel, about 20 cm away from the bed, held for 5  seconds, put down for 5 seconds, repeat 10 times |
| --- | --- |
| 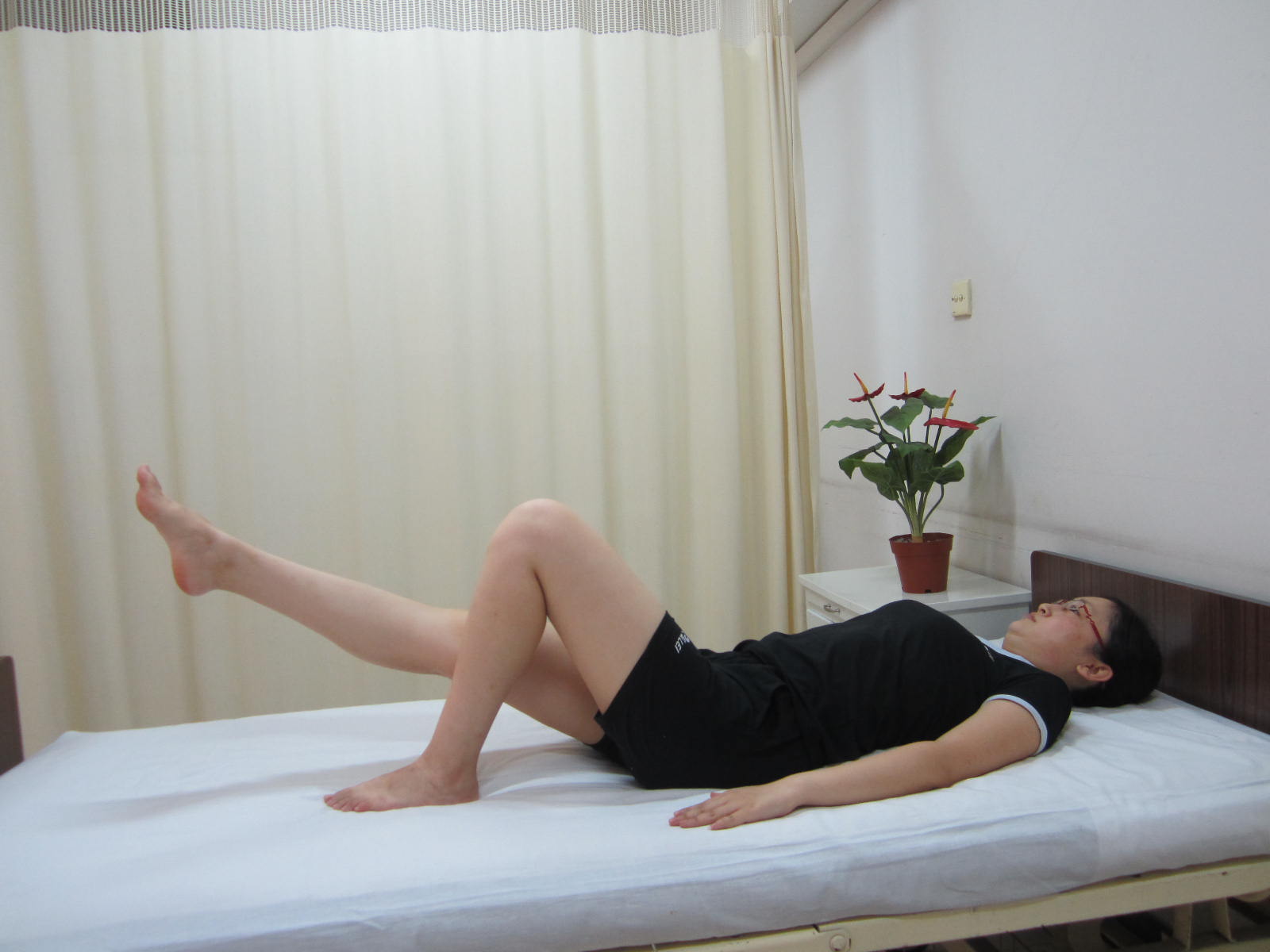③Relax this leg and repeat the above action on the other side | 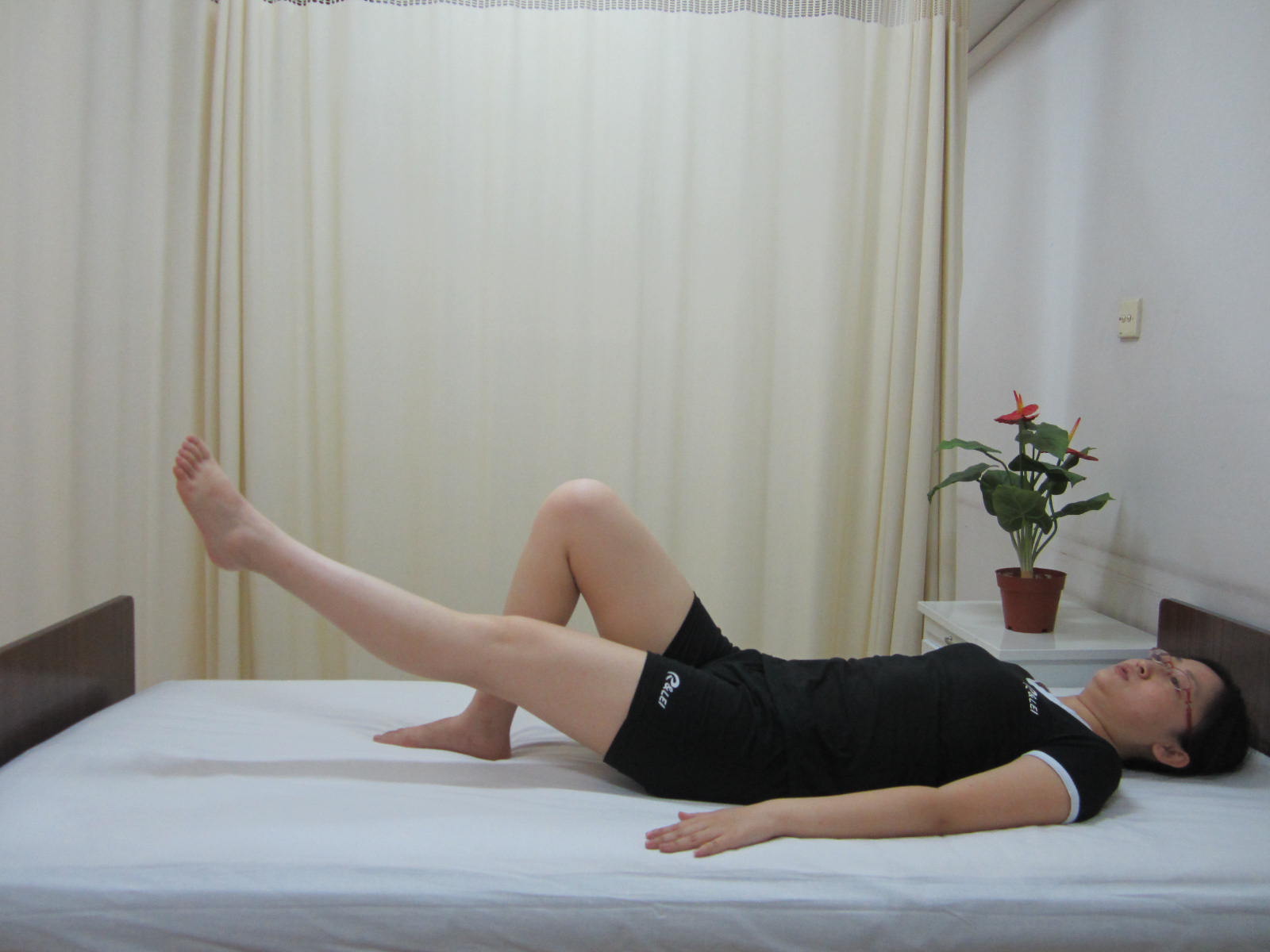  ④Exercise alternately 3 to 5 times with both legs |

M-3：Leg lifts in the prone position

| 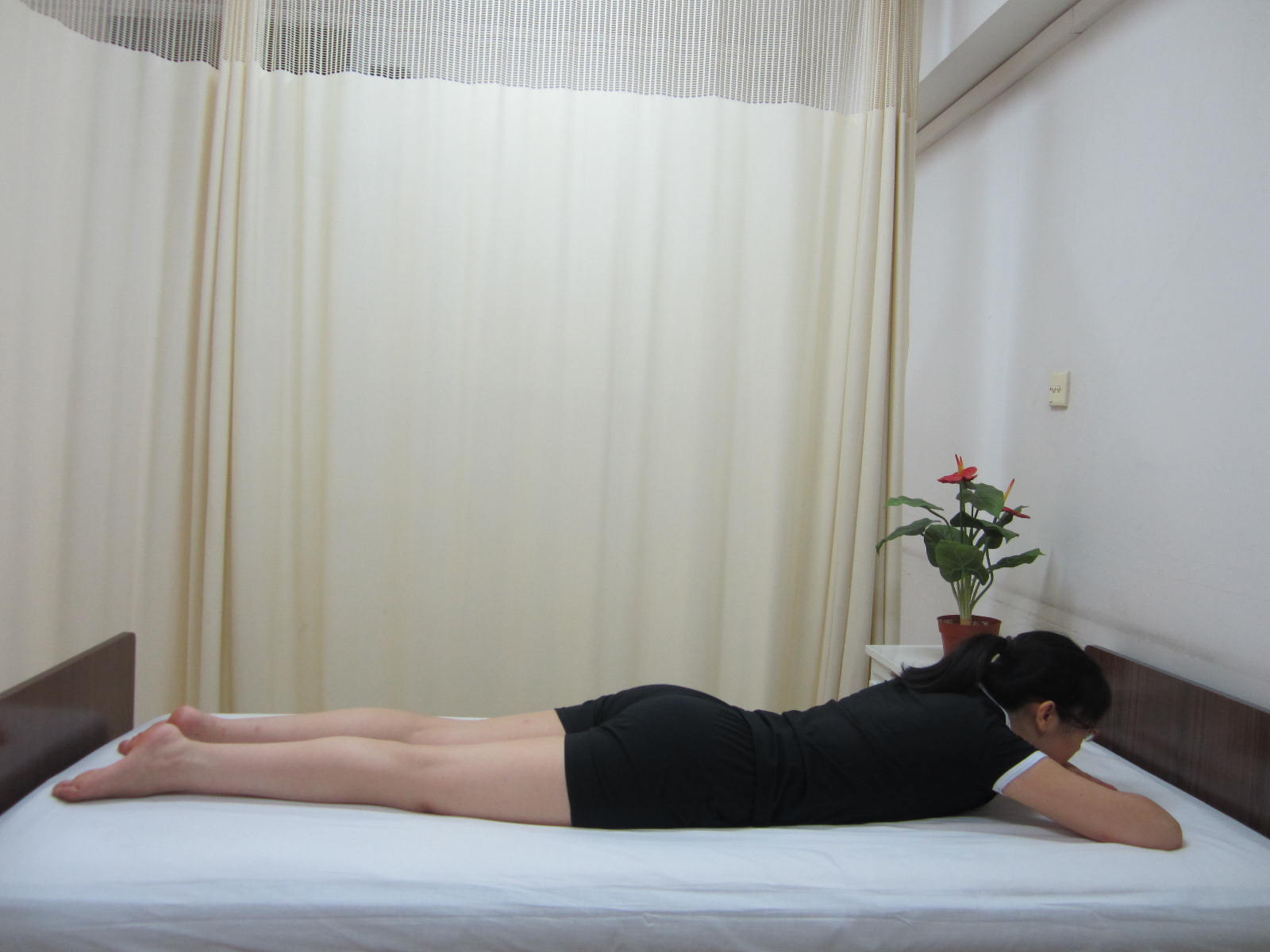  ①Lie face down, stretch knees | 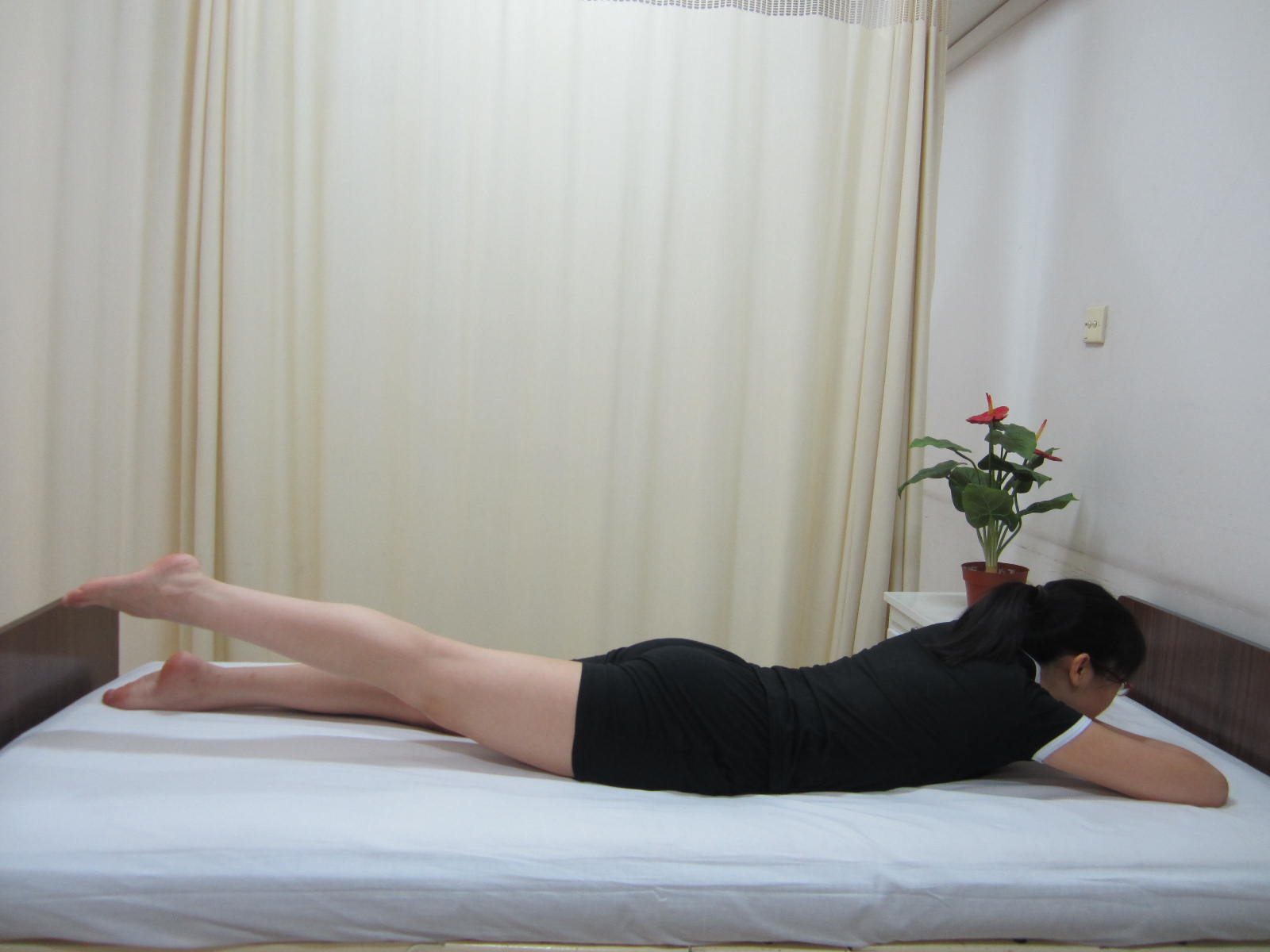  ②Lift one leg back to the toe, about 20 cm away from the bed, held for 5 seconds, put down for 5 seconds, repeat 10 times |
| --- | --- |
| 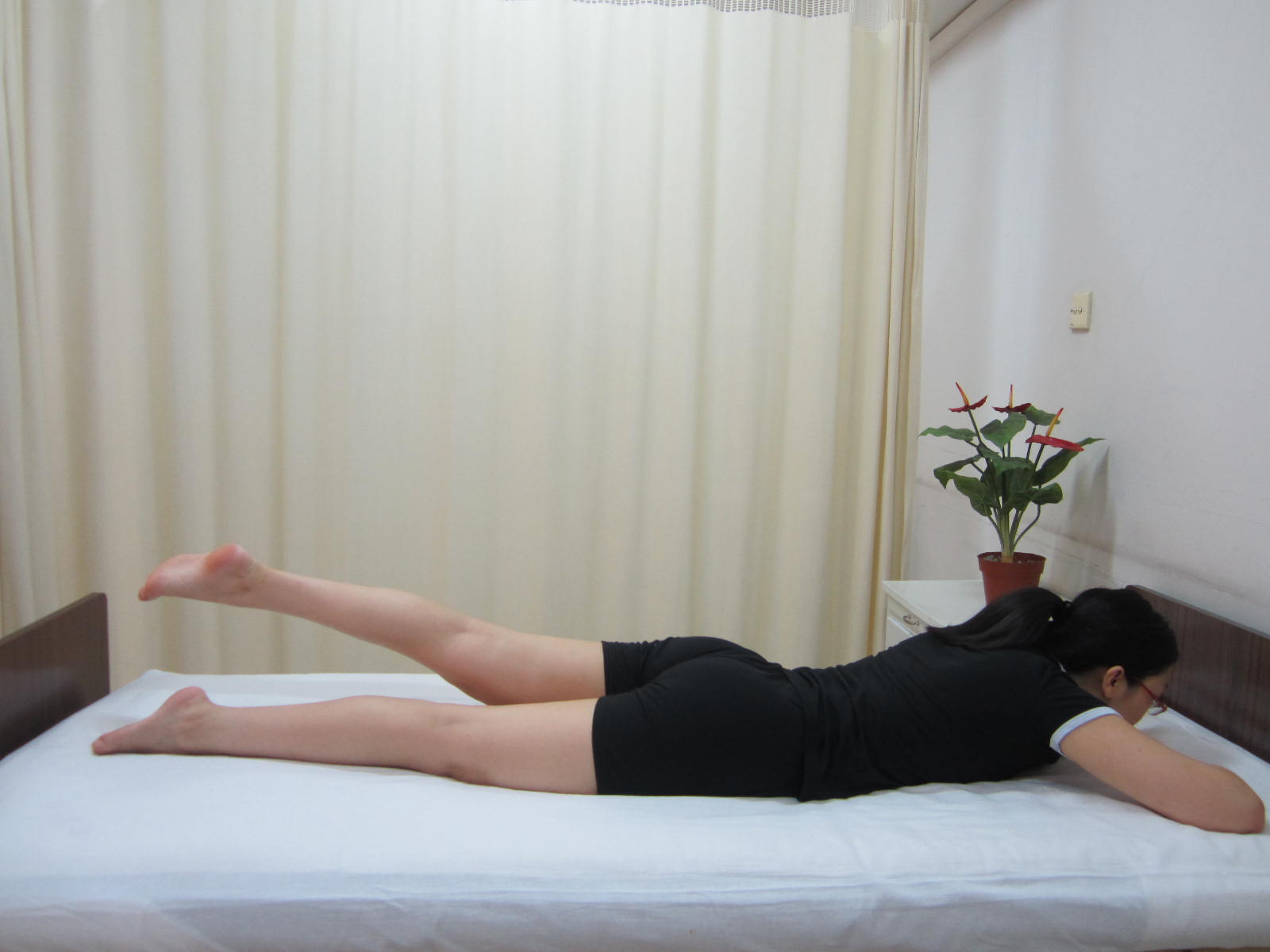③Relax this leg and repeat the above action on the other side | 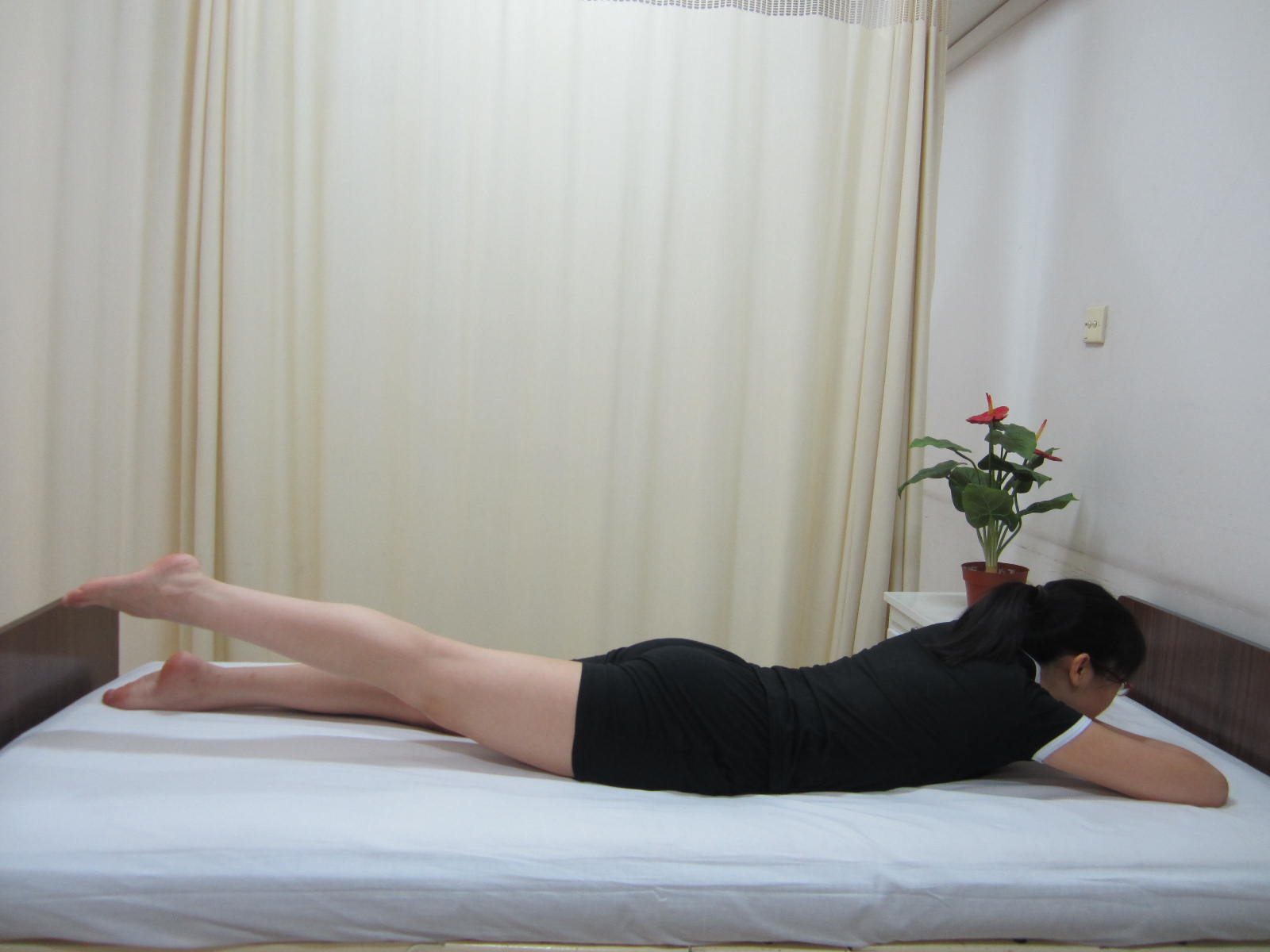  ④Exercise alternately 3 to 5 times with both legs |

B-1：Shifting the center of gravity (left and right)

| 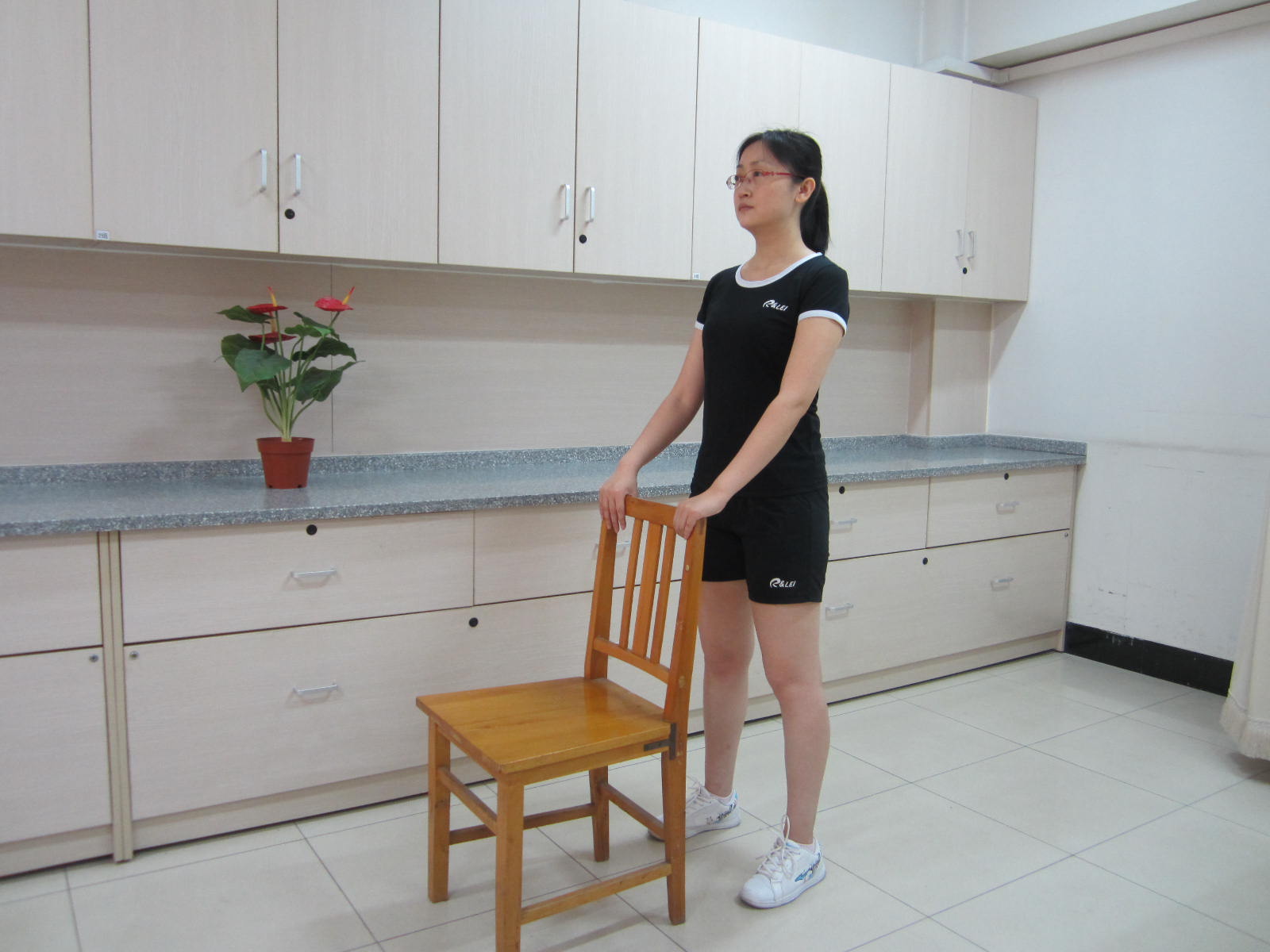  ①Stand up and support a chair with a height of 70~80 cm and open the feet | 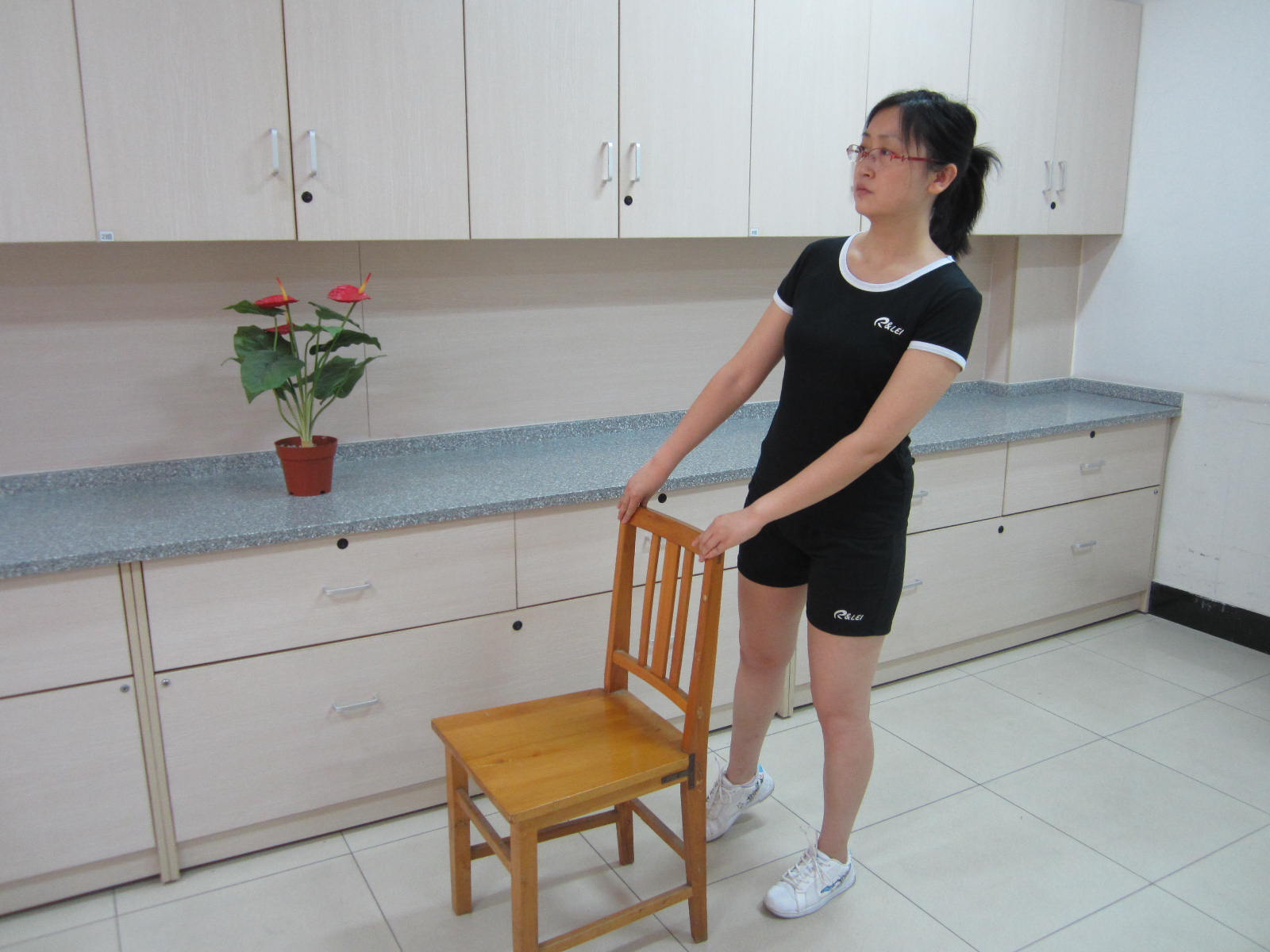  ②Keep your knees upright, slowly move the center of gravity to the left, and gradually lower your right heel |
| --- | --- |
| 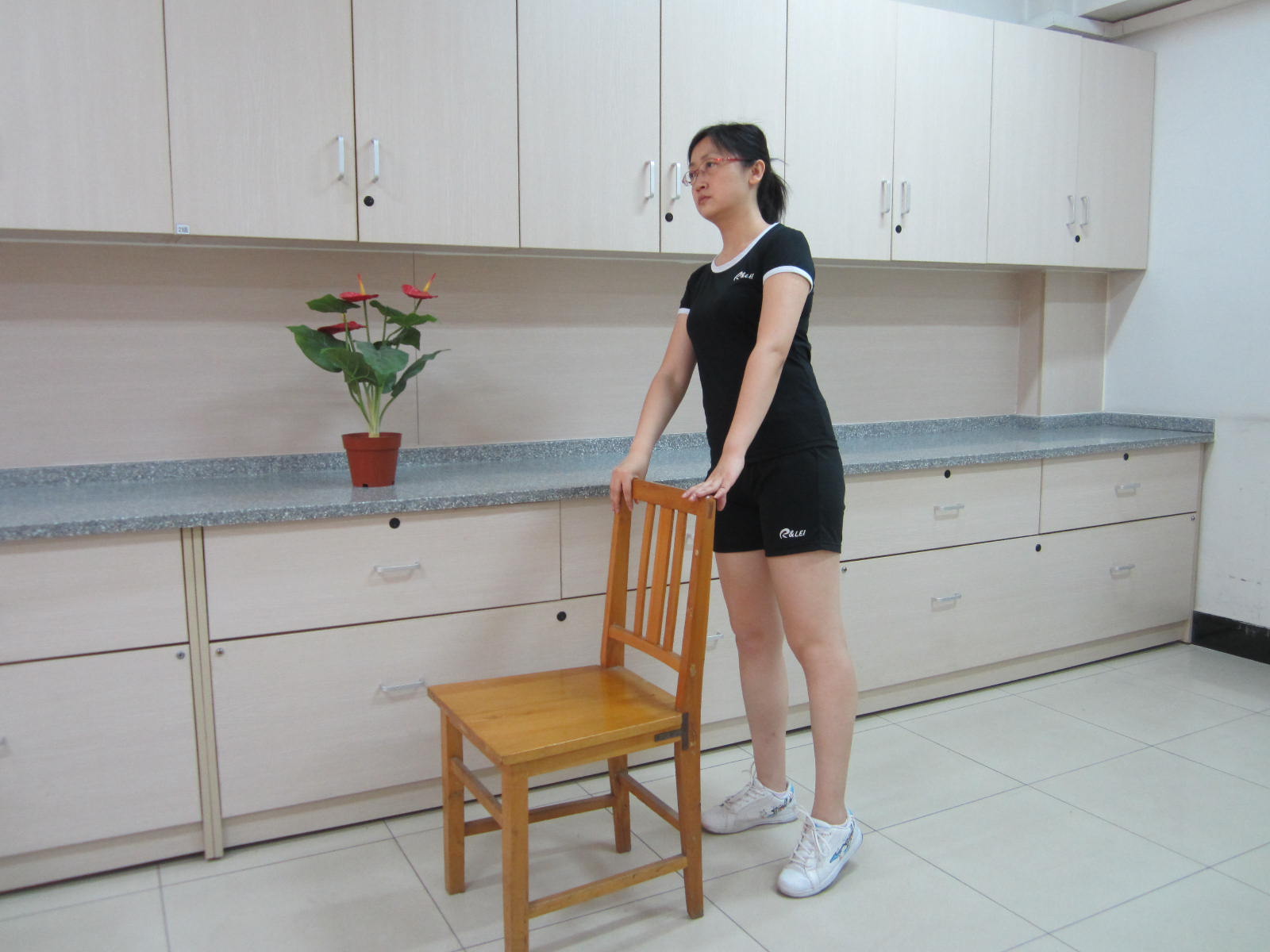  ③Keep your knees upright, slowly move the center of gravity to the right, and gradually lower your left heel | 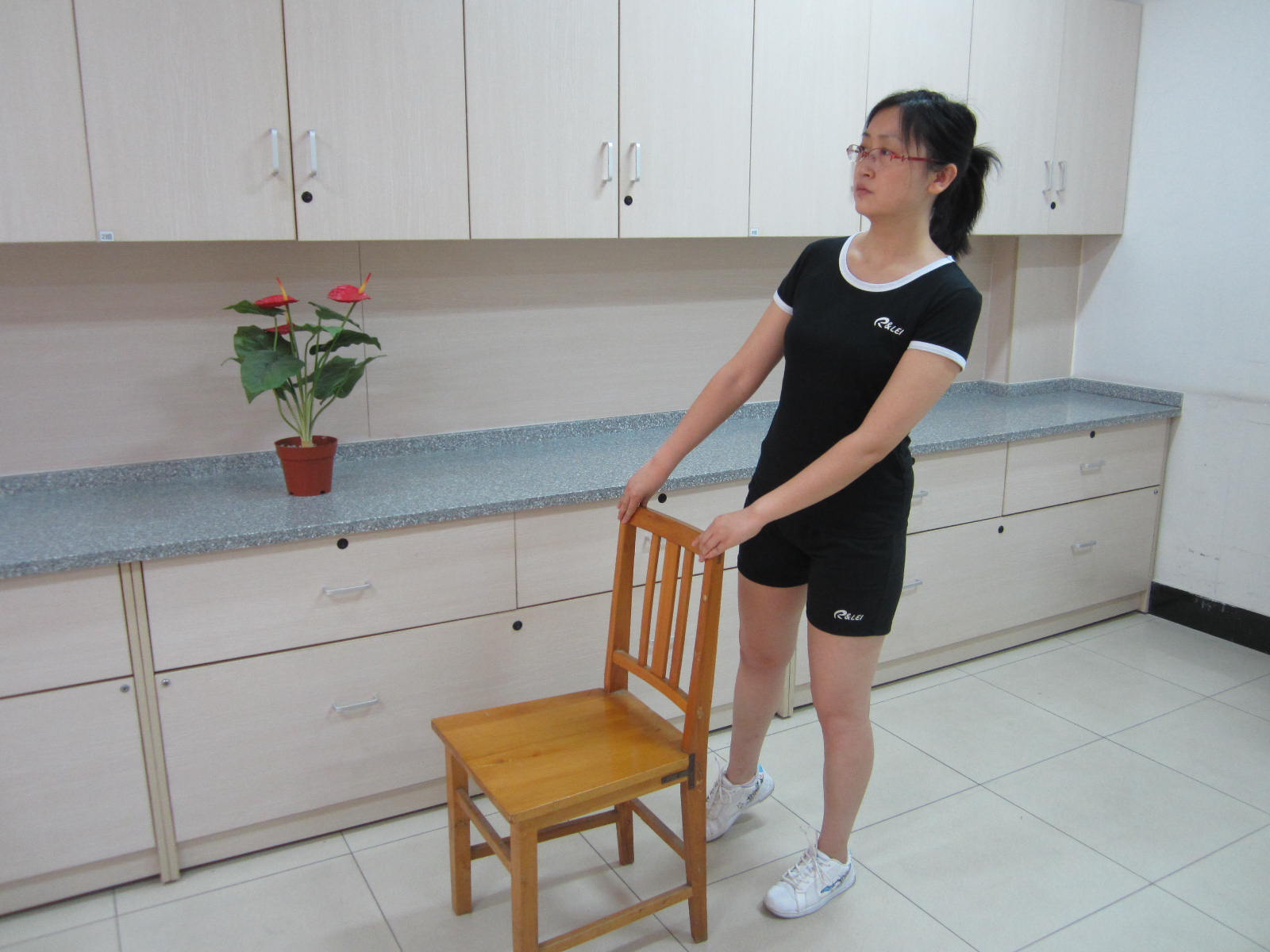  ④Repeat the above action for 3 minutes |

B-2：Shifting the center of gravity (before and after)

| 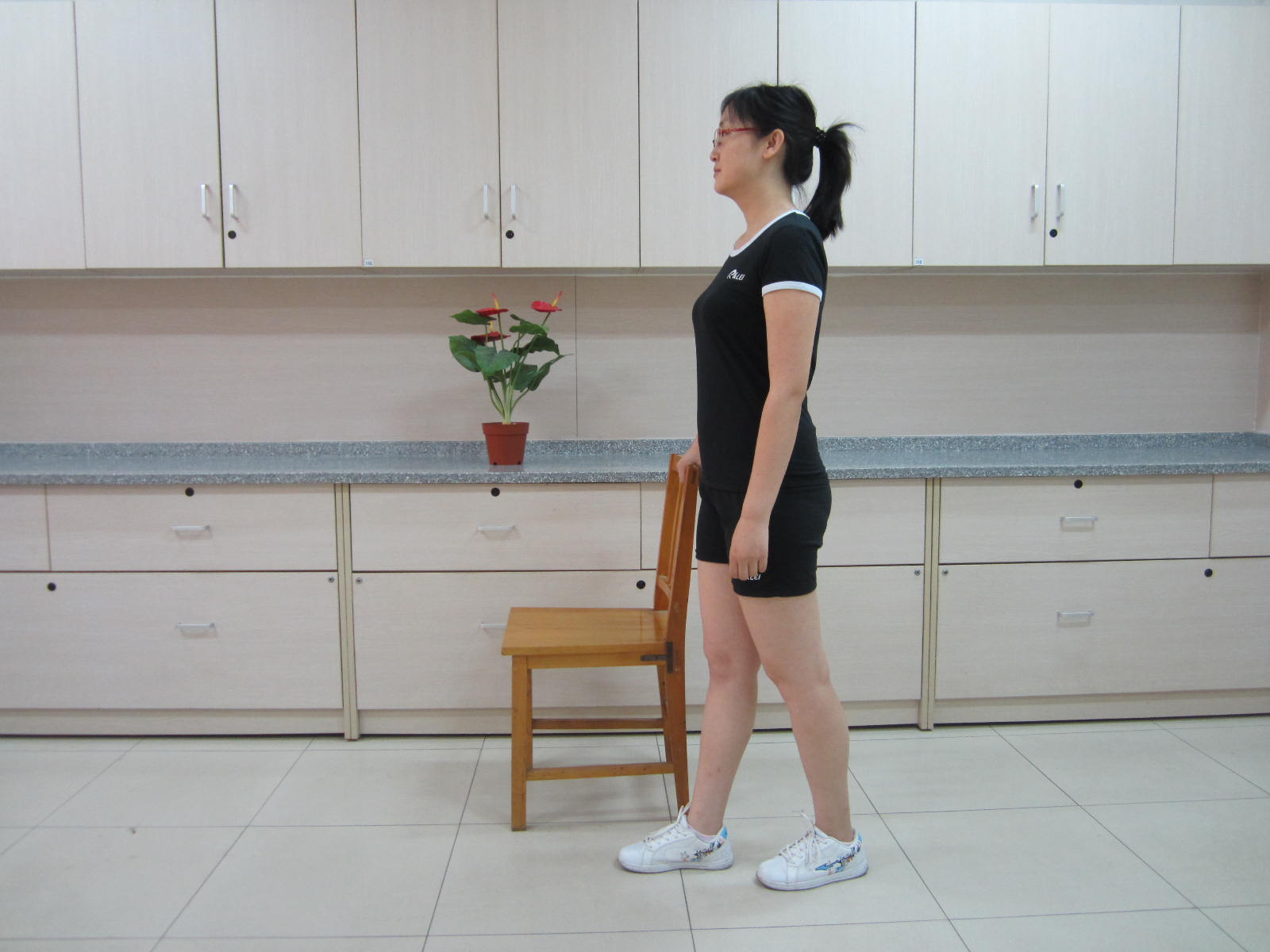  ①Stand up and support a chair with a height of 70~80 cm and take one step forward on one side | 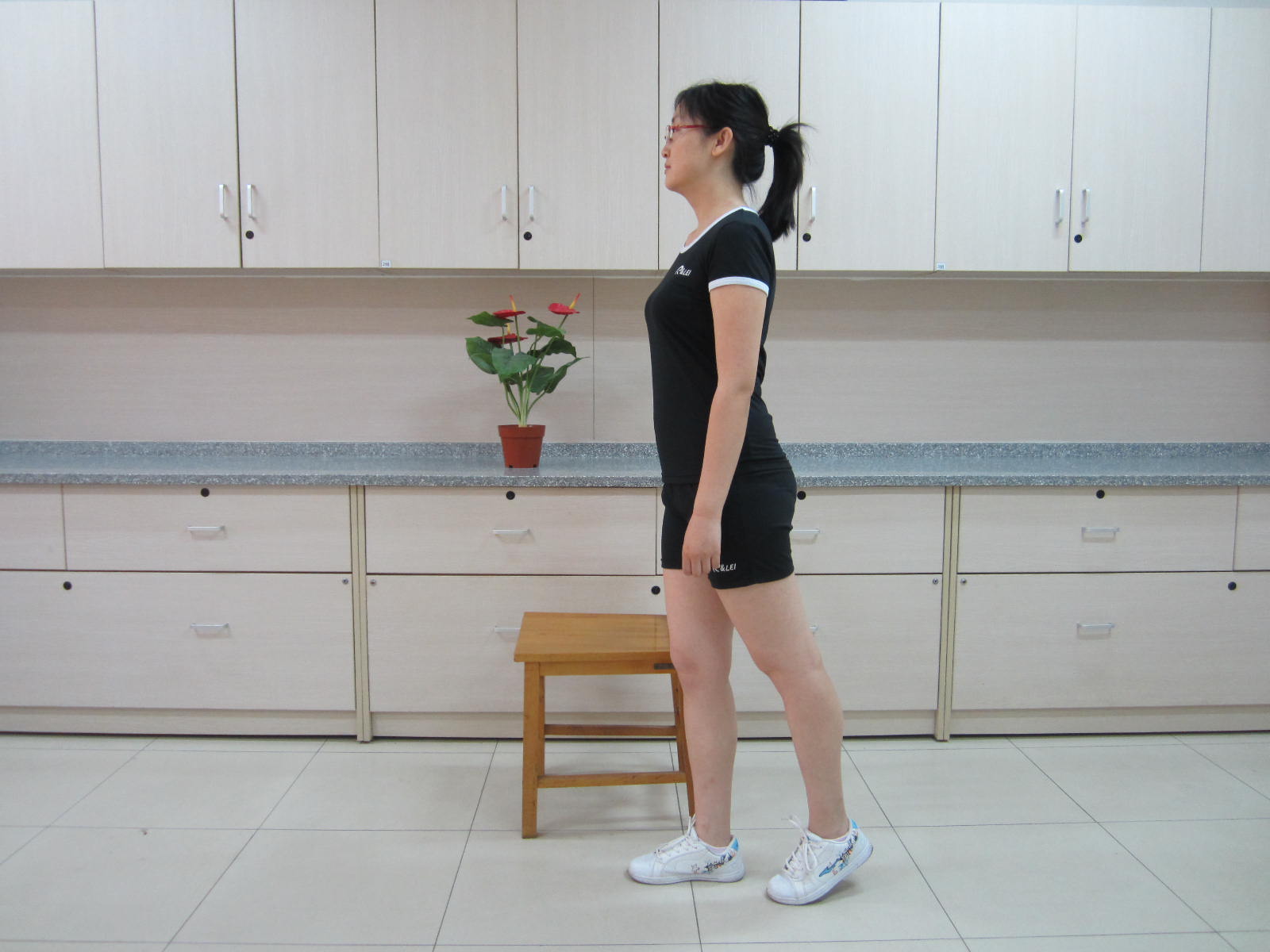  ②Keep the knees upright, slowly move the center of gravity forward, and the heel of the hind foot gradually leaves the ground |
| --- | --- |
| 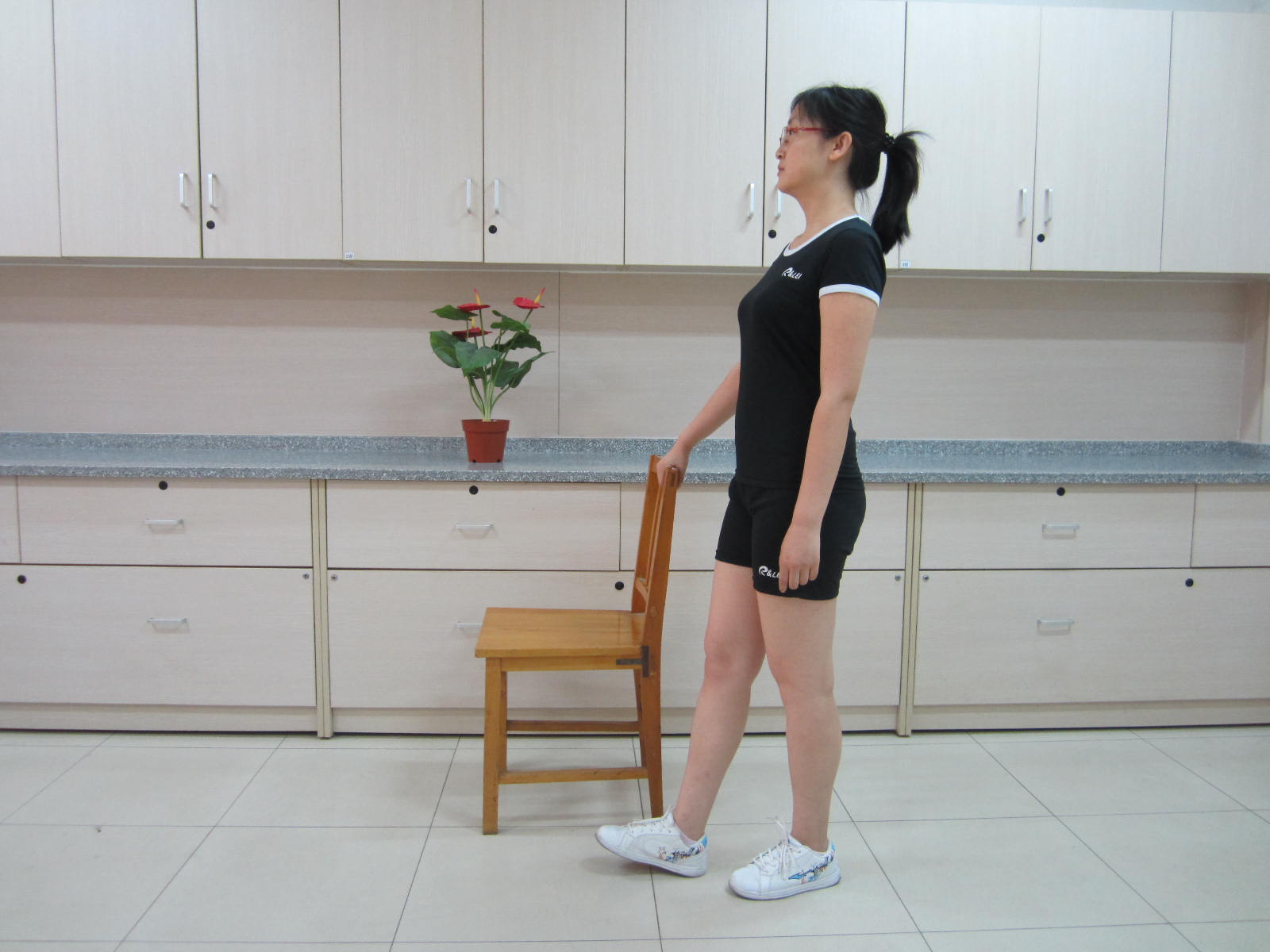  ③Keep the knees upright, slowly move the center of gravity backwards, and the forefoot gradually leaves the ground | 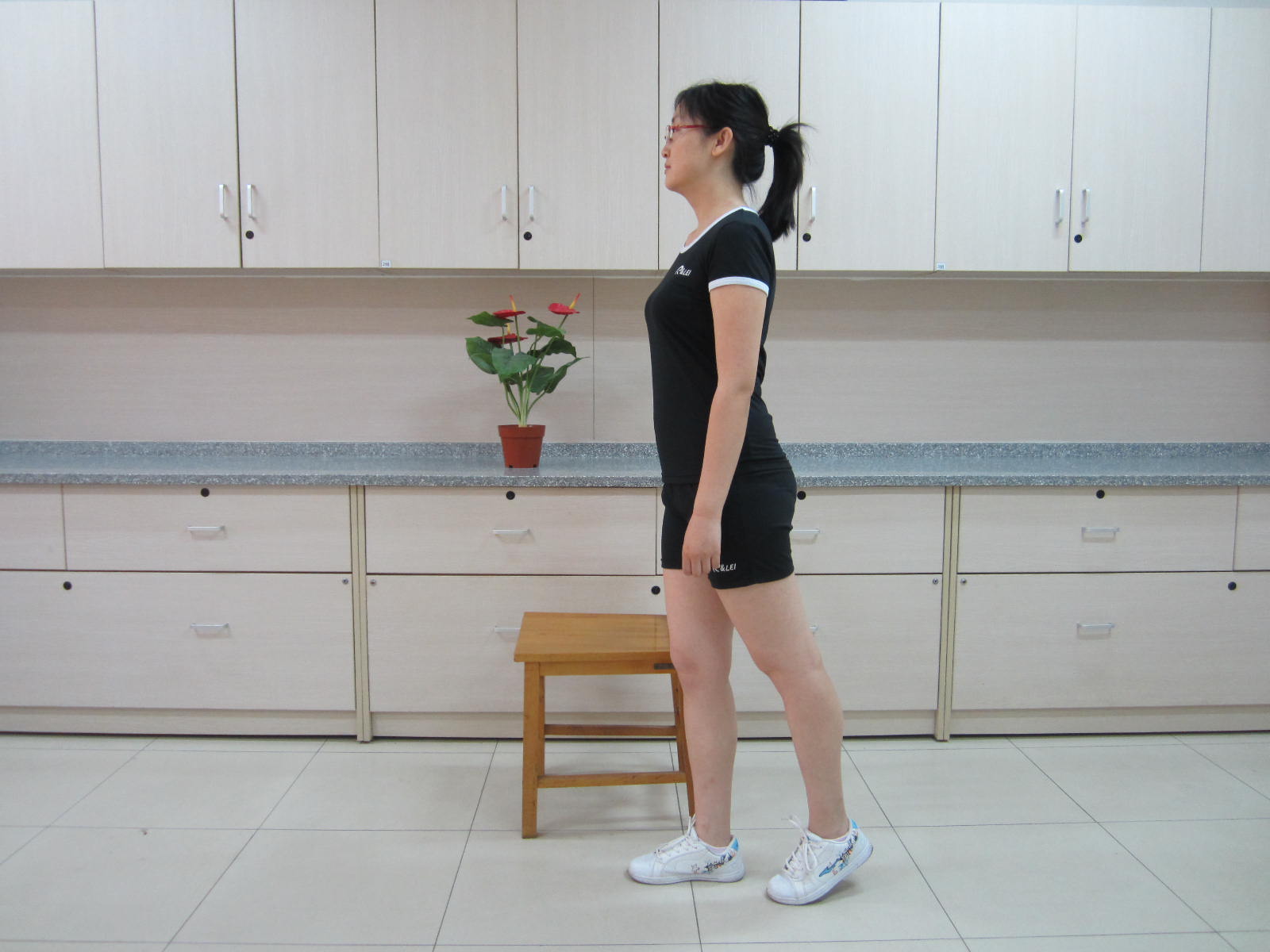  ④Repeat the above action for 3 minutes |

M-4：Resistance knee extension

| 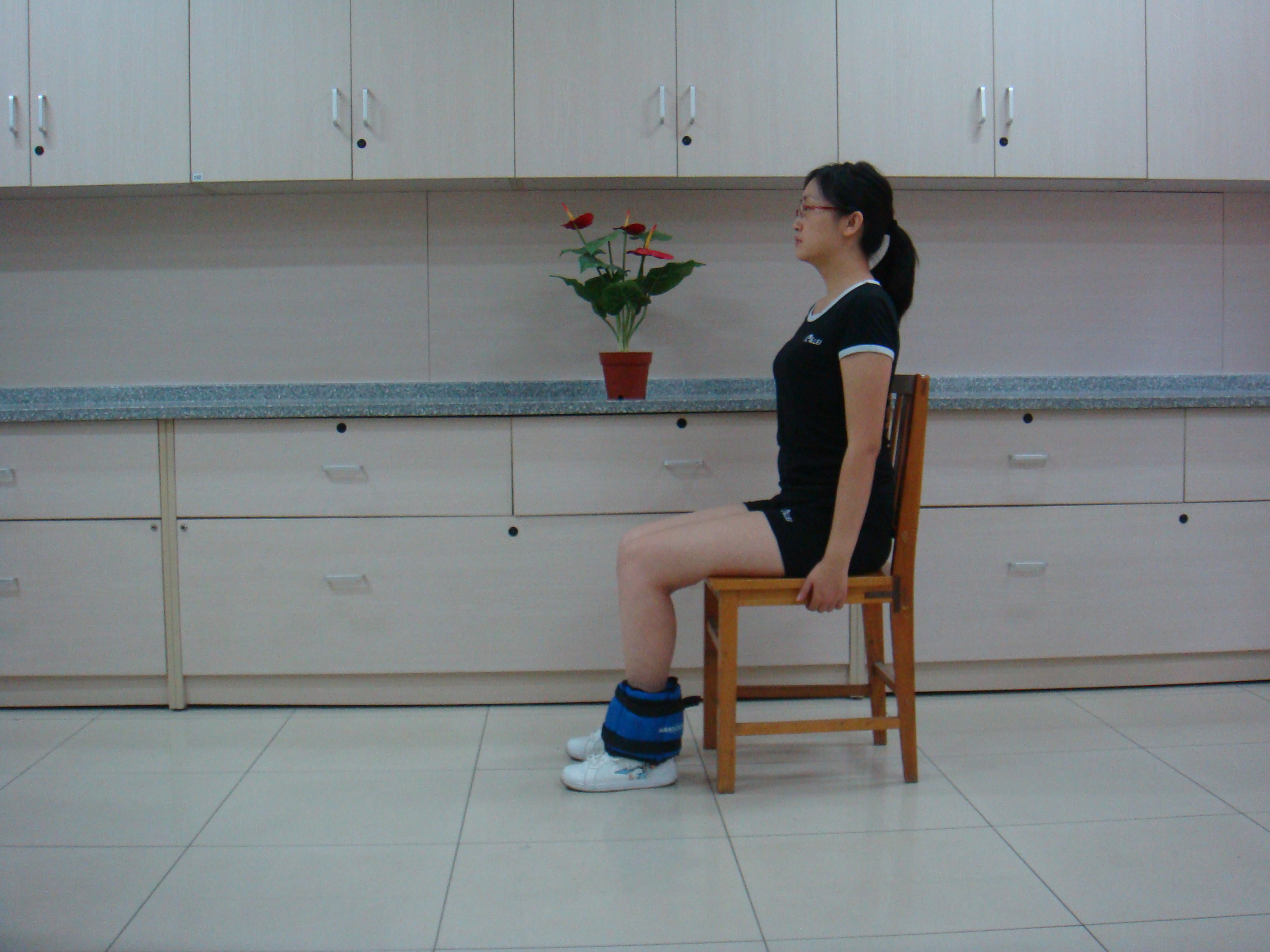  ①Sit on the chair or at the bed, tie a 1kg sandbag to the ankle, keep the upper body straight | 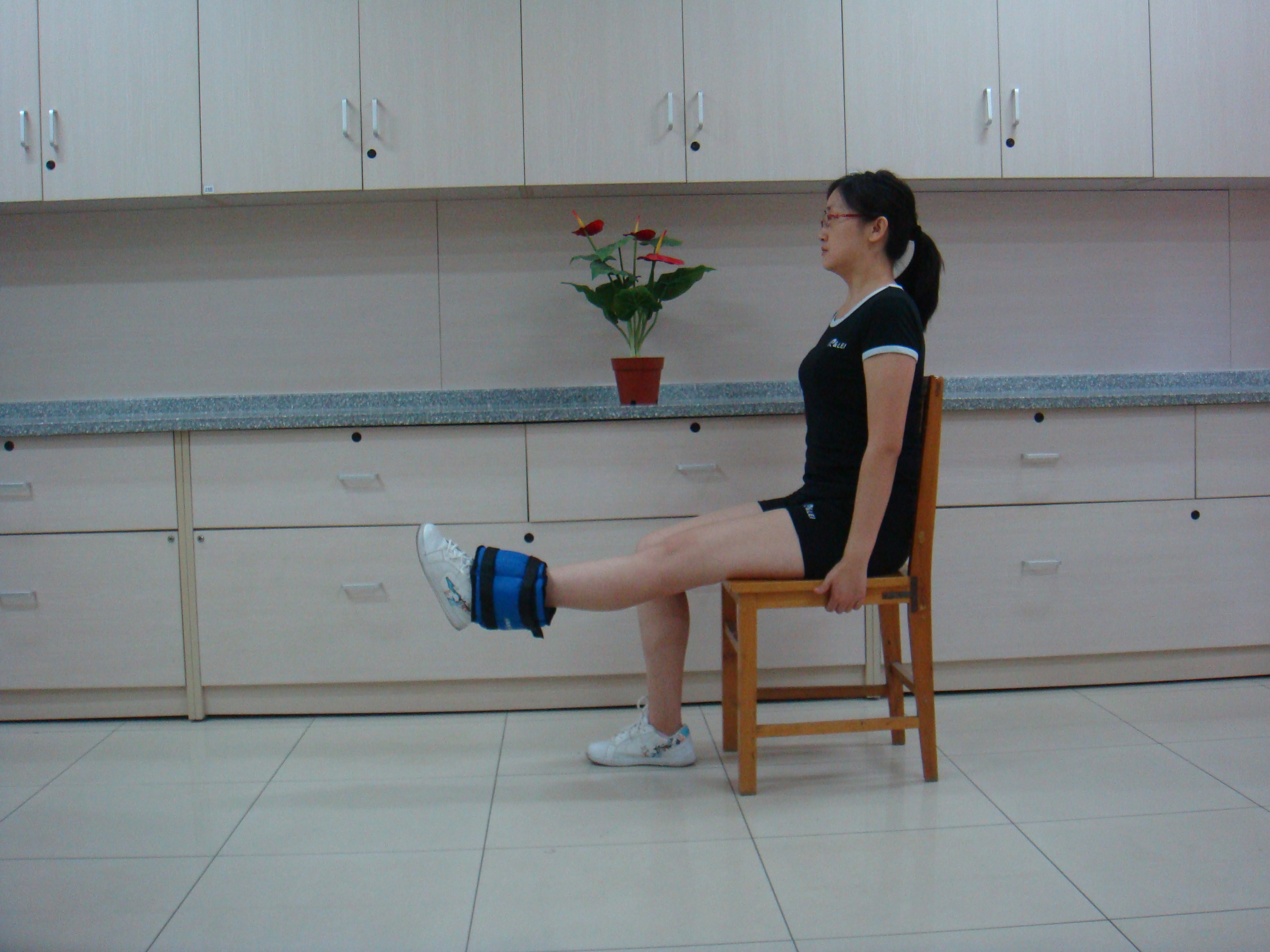  ②Do not move the thighs, lift your calves until the knees are fully extended, hold for 5 seconds, rest your legs for 5 seconds, repeat 10 times |
| --- | --- |
| 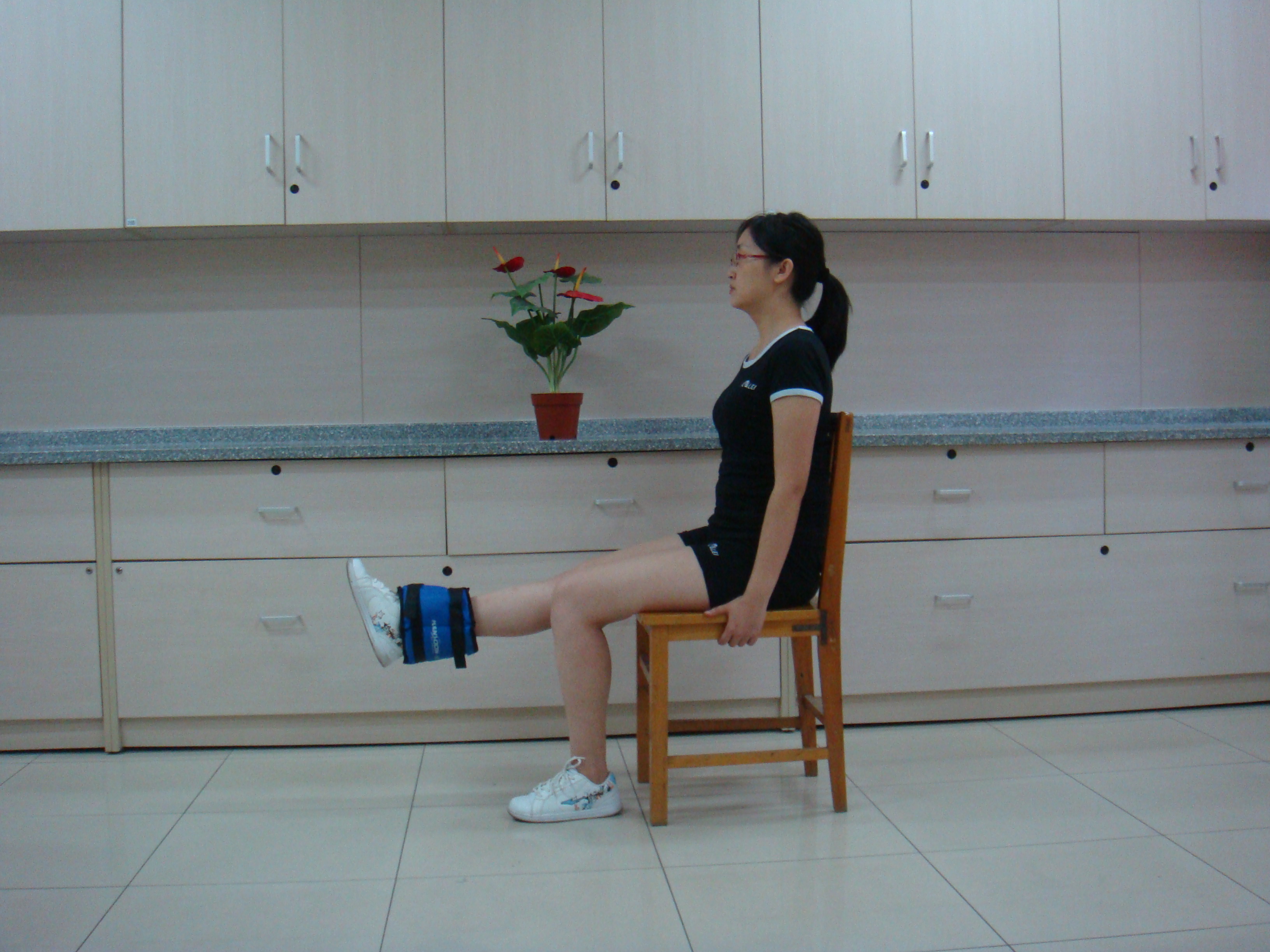③Relax this leg and repeat the above action on the other side | 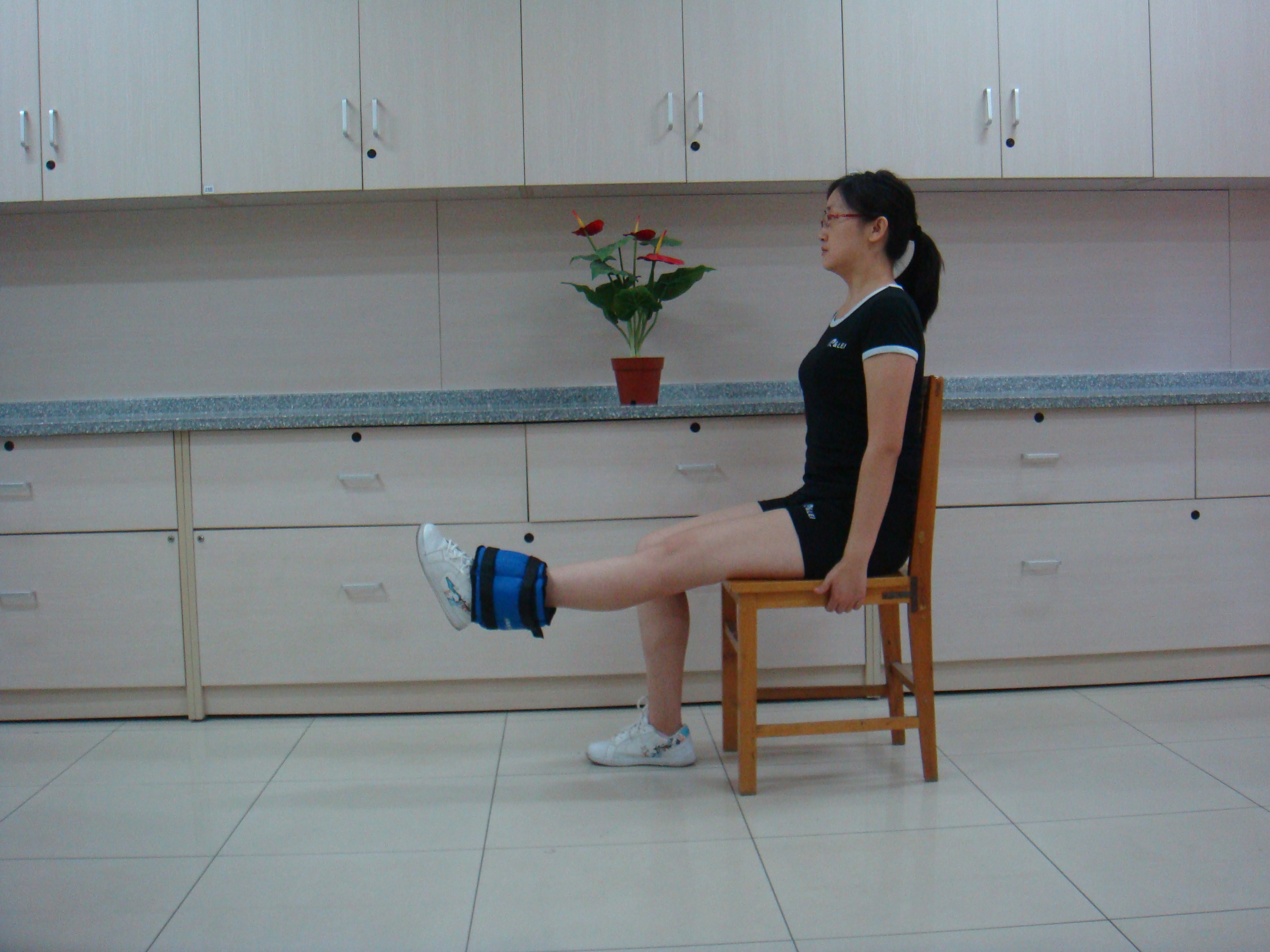  ④Exercise alternately 2 to 3 times with both legs |

M-5：Resistance knee flexion

| 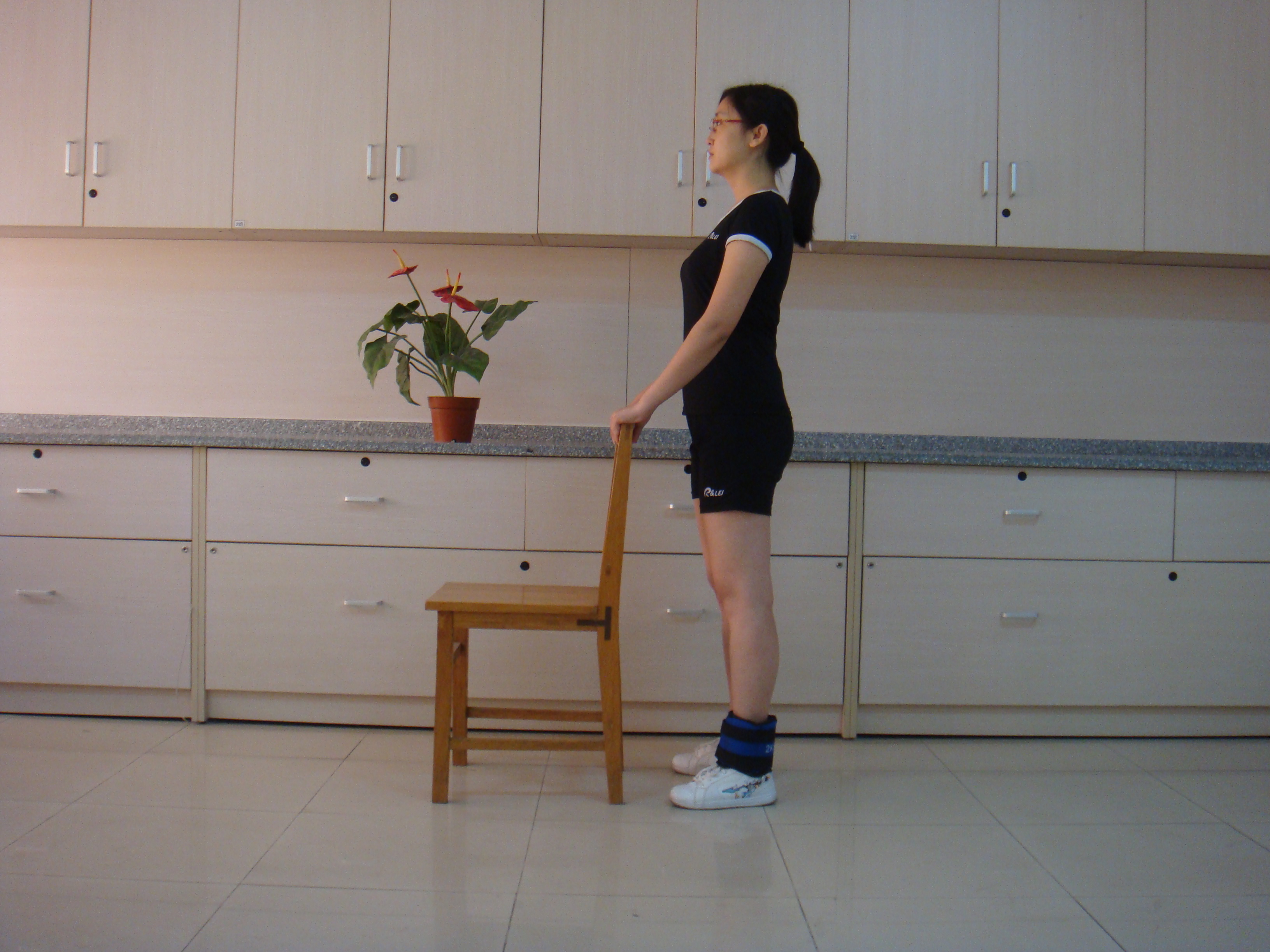① Standing up, tie a 1kg weight sandbag to the ankle joint, and support the upper edge of the chair | 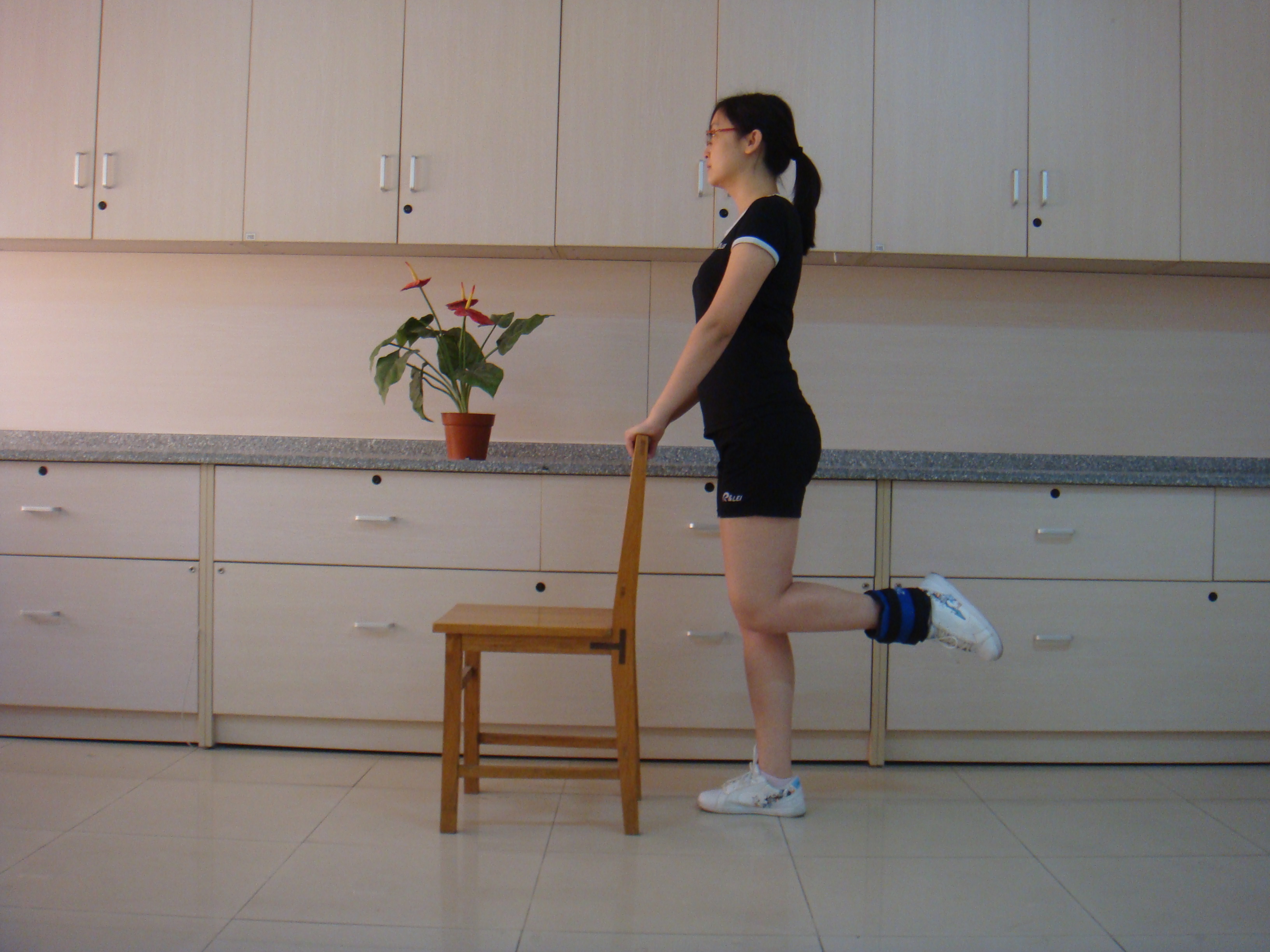② Stand on one leg and pull the calf back to the other leg, flexing the knee as much as possible while keeping the thigh perpendicular to the ground. Hold for 5 seconds, put your legs down for 5 seconds, repeat 10 times |
| --- | --- |
| 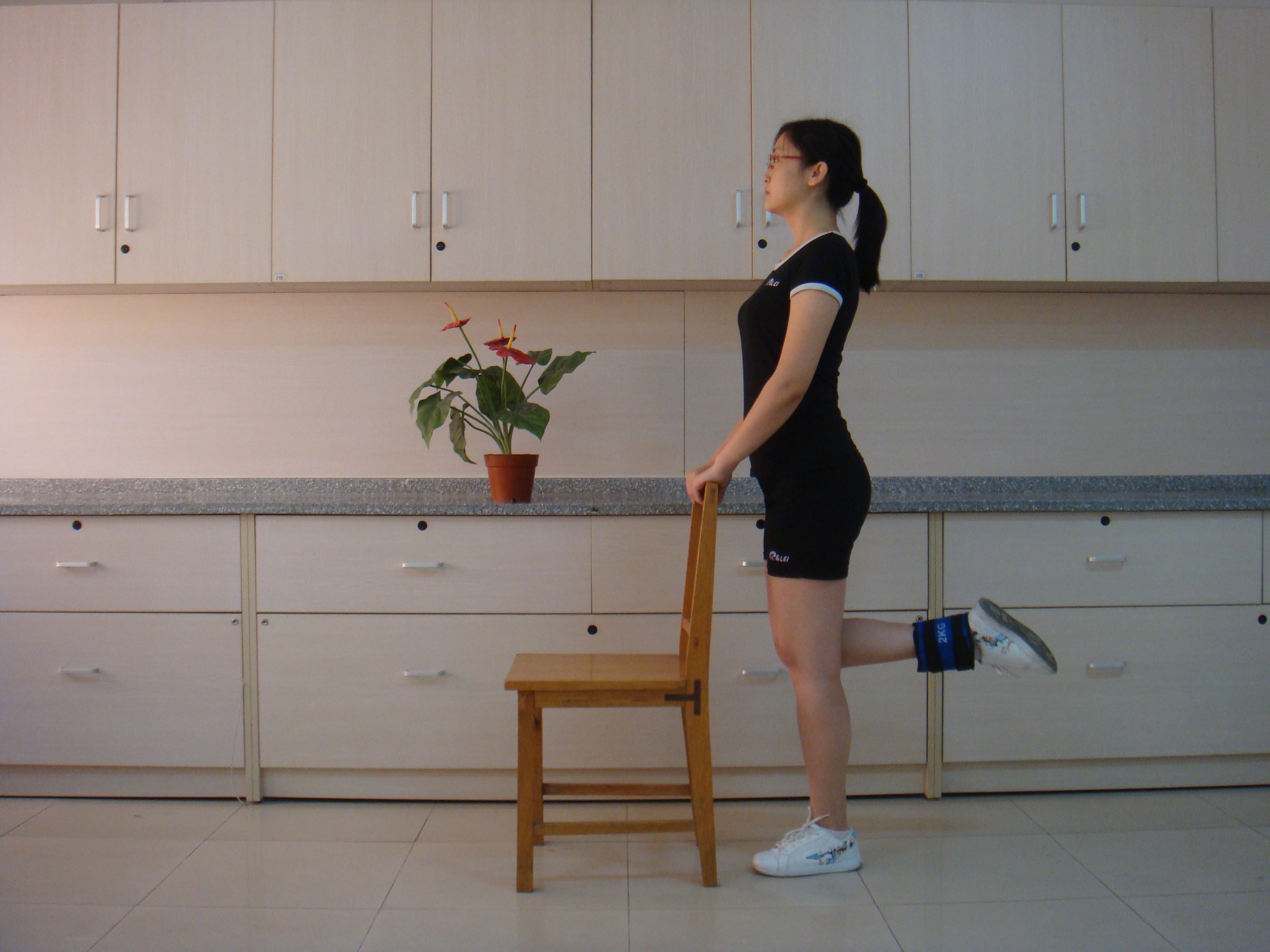③ Relax this leg and repeat the above action on the other side | 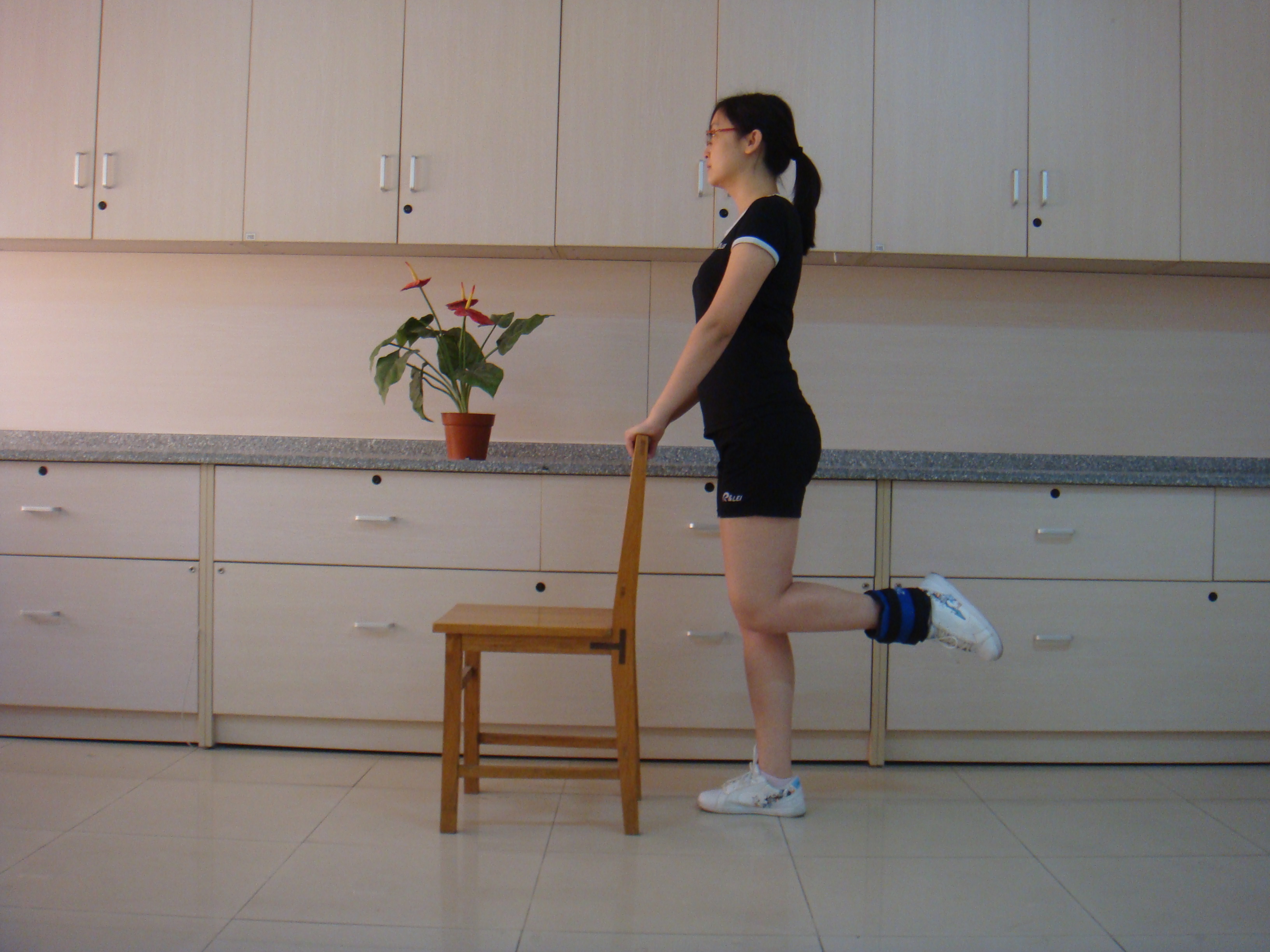④ Exercise alternately 2 to 3 times with both legs |

**Precautions:**

1. **Safety first:** Practice after professional guidance. It is better to have family members to accompany you.

2. **Integrate exercise into daily life:** Exercise while watching TV, pay attention to natural breathing, don't hold your breath

3. **Appropriate amount:** The action should be easy and smooth. If pain or discomfort is aggravated, it may be caused by excessive or excessive movements. The amplitude and number of exercises can be appropriately reduced. When the symptoms are obviously aggravated, stop practicing and consult a professional.

**
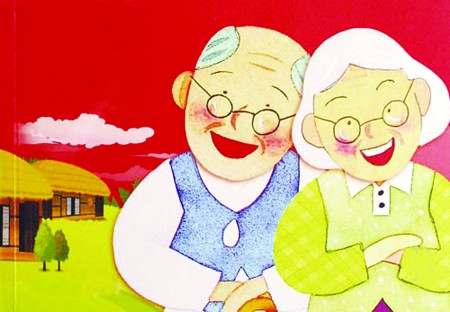
**

**Wish you a happy life!**
